# Single-Cell Census of Mechanosensitive Channels in Living Bacteria

**DOI:** 10.1371/journal.pone.0033077

**Published:** 2012-03-13

**Authors:** Maja Bialecka-Fornal, Heun Jin Lee, Hannah A. DeBerg, Chris S. Gandhi, Rob Phillips

**Affiliations:** 1 Biochemistry and Molecular Biophysics Option, California Institute of Technology, Pasadena, California, United States of America; 2 Department of Applied Physics, California Institute of Technology, Pasadena, California, United States of America; 3 Department of Physics and the Center for Physics of Living Cells, University of Illinois at Urbana-Champaign, Urbana, Illinois, United States of America; 4 Division of Chemistry and Chemical Engineering, California Institute of Technology, Pasadena, California, United States of America; 5 Division of Biology, California Institute of Technology, Pasadena, California, United States of America; University of Groningen, Netherlands

## Abstract

Bacteria are subjected to a host of different environmental stresses. One such insult occurs when cells encounter changes in the osmolarity of the surrounding media resulting in an osmotic shock. In recent years, a great deal has been learned about mechanosensitive (MS) channels which are thought to provide osmoprotection in these circumstances by opening emergency release valves in response to membrane tension. However, even the most elementary physiological parameters such as the number of MS channels per cell, how MS channel expression levels influence the physiological response of the cells, and how this mean number of channels varies from cell to cell remain unanswered. In this paper, we make a detailed quantitative study of the expression of the mechanosensitive channel of large conductance (MscL) in different media and at various stages in the growth history of bacterial cultures. Using both quantitative fluorescence microscopy and quantitative Western blots our study complements earlier electrophysiology-based estimates and results in the following key insights: i) the mean number of channels per cell is much higher than previously estimated, ii) measurement of the single-cell distributions of such channels reveals marked variability from cell to cell and iii) the mean number of channels varies under different environmental conditions. The regulation of MscL expression displays rich behaviors that depend strongly on culturing conditions and stress factors, which may give clues to the physiological role of MscL. The number of stress-induced MscL channels and the associated variability have far reaching implications for the *in vivo* response of the channels and for modeling of this response. As shown by numerous biophysical models, both the number of such channels and their variability can impact many physiological processes including osmoprotection, channel gating probability, and channel clustering.

## Introduction

The connection between structure and function is one of the key tenets of modern biology. However, structure alone cannot explain the physiological workings of a given organism. A more nuanced view takes into account the single-cell numbers, stoichiometry, and population distribution of the various molecules that partake in the life processes of an organism. As a result of this challenge, recent years have seen a number of careful studies aimed at establishing the census of the molecular actors in a host of different situations. Examples include the distribution of metabolites in bacteria [1], the distribution of proteins related to the actin cytoskeleton [2], counts of the proteins involved in glycolysis in yeast [3] and the stoichiometry of proteins associated with adhesion complexes [4]. Similar studies have been made that attempt to provide a genome-wide snapshot of the distribution of mRNAs or proteins (or in some cases both) [5], [6], [7], [8], [9]. This partial accounting only scratches the surface of efforts currently underway to measure the number of molecules present in a cell at different stages in the cell cycle and in response to various environmental insults. To date, this kind of molecular census taking has yet to be performed on proteins related to mechanosensation.

Mechanosensation is a key biological process found across all domains of life and over a wide range of spatial and energy scales [10]. One important case study in mechanosensation is provided by mechanosensitive channels in bacteria [11]. One of them, the mechanosensitive channel of large conductance (MscL), has been studied extensively, both using crystallography to provide structural snap shots [12], [13] and single-channel recordings to measure the gating properties of this channel [14], [15]. Despite these numerous studies, the *in vivo* characterization of MscL expression levels under physiological conditions remains relatively limited. In previous work, it was found that the relative mean abundance of mechanosensitive channels increased under stress conditions such as starvation and high media osmolality [16]. We set out to undertake a molecular census of MscL channels expressed in single *Escherichia coli* cells under various growth and stress conditions with an absolute number calibration. We also sought to measure the cell to cell variability and to assign functional forms to the population distributions.

The absolute mean number of channels is a vital “conversion” parameter which can bridge the single-molecule level understanding resulting from electrophysiological measurements, X-ray structures and biophysical models to the ensemble phenotypic behavior of MscL. Single-channel electrophysiology provides the single-channel conductance of an open MscL channel and the probability that the channel is open under any given tension, but these data have rarely been translated into the behavior in the cell. The mean number of channels per cell, in principle, allows an extrapolation of the single channel results to a whole cell (with certain exceptions noted below) and can determine the total number of channels open and how much total transport occurs. That is, a proper molecular census can make single-molecule results relevant to the *cellular* scale. We note, however, there are instances of MscL mutant channels that exhibit behavior in electrophysiology that are different from the properties expected from cell-based assays [17], [18].

A paradigmatic view of these channels is that they serve as osmotic relief valves when microbes are subjected to osmotic shock [15], [19]. Currently, it is unclear just how many channels are needed for osmotic protection or how much total water transport occurs during osmotic shock. Biophysical models [20], [21] that estimate the magnitude of these effects depend on the mean number of channels and can vary according to the assumed expression level. Another interesting biophysical example is related to cooperative gating [22] and clustering [22], [23] where each phenomenon is predicted to be strongly dependent on the areal density of channels.

Previous estimates of the number of channels per cell were based on radio-labeling of purified membrane fractions [24] or electrophysiology studies performed on giant spheroplasts [11], [16], [25] and are not truly, or at least directly, *in vivo*. They can be affected by assumptions of the reconstituted protein density, geometric scaling of the patch, and/or number of cells needed to form a spheroplast. These results are summarized in Table 1, showing a range of 4 to 100 conducting channels per cell. The uncertainty of these estimates prevents us from quantitatively relating the single-molecule and phenotypic pictures and, as such, motivates a more definitive measurement. Further, these assays, at best, can estimate the total number of active channels. We do not know how many channels are inactive or have been inactivated, either *in vivo* and/or after preparation for *in vitro* measurements. In this work we present an absolute MscL census *in vivo*, which eliminates uncertainties related to reconstitution and paves the way for a measurement of the total number of channels, both active and inactive.

**Table 1:** Summary of reports on the number of MscL channels per *E. coli* cell.

Number per Cell	Method	Reference
50	radiolabeling	[24]
10–100	electrophysiology	[11]
4–5	electrophysiology	[16]
10–15	electrophysiology	[25]

Additionally, the mean number of channels may change with growth conditions. We postulate that shifts in the population census in response to environmental factors yields an improved contextual understanding of the physiological role of MscL and the factors that control its induction and regulation. Previous work [16] found that production of MS channels is induced by entry into stationary phase or by growth in a media supplemented with NaCl to increase its osmolality. These studies also demonstrated that MscL expression is enhanced by the production of the sigma factor RpoS (also known as σ^s^), the primary sigma factor controlling regulation during stress response or in preparation for stationary phase [26]. There are numerous types of stress responses associated with RpoS [27], well beyond the scope of this work to consider in a comprehensive way. However, one particularly intriguing response is related to the carbon source of the media. Media associated with slower growth rates have been observed to induce elevated RpoS levels [28], [29], [30]. Thus, different types of culture media may offer a means of inducing different levels of RpoS and associating MscL with functions other than osmotic protection. As a result, we examine the expression levels of MscL and RpoS in three common media with progressively slower growth rates: the Miller variant of LB media with 10 g/L NaCl (LB-Miller) [31], M9 minimal media supplemented with glucose (M9+glucose), and M9 media supplemented with glycerol (M9+glycerol). We also measure the cell-to-cell variability in expression level. The population distribution of the number of protein subunits is a quantity of great interest and recent insights [32], [33], [34], [35], [36] make it possible to relate the characteristics of a distribution to the “noise” associated with the processes of both transcription and translation, which, in turn, provides a means of probing the underlying regulatory architecture.

Our approach presented in this study is complementary to earlier electrophysiological studies. We measure the mean number of MscL channels per cell under various culturing conditions using quantitative Western blots and single-molecule calibrated fluorescence microscopy. Further, by measuring the fluorescence of thousands of individual cells expressing chromosomally-integrated MscL fluorescent protein fusions, we determine not only the mean number of channels per cell, but also the cell-to-cell variability.

## Results

To carry out the measurements described above, two independent techniques were employed. We used fluorescence microscopy to measure the distribution of MscL-sfGFP expression levels across a population of cells subjected to different growth and stress conditions. In parallel, we performed a series of bulk assays using quantitative Western blots to compare the mean expression levels of MscL-sfGFP to wild-type MscL to ensure that the GFP fusion does not alter native expression levels.

### Confirming Native Expression and Proper Function of MscL-sfGFP Fusion Proteins

MscL-fluorescent protein fusions have been previously expressed in *E. coli* using exogenous plasmids with inducible promoters [37]. However, our experiments require MscL fusion proteins produced at native levels, where the expression level is controlled by the endogenous MscL regulatory system. To accomplish this, we replaced the native MscL coding region of a wild-type *E. coli* strain (MG1655) with a sequence that coded for MscL fused to super-folder green fluorescent protein (sfGFP) [38] (see *Chromosomal Integration* in Materials and Methods), creating the MscL-sfGFP fusion expressing strain MLG910. To compare the expression levels between the MLG910 and MG1655 strains, we performed a series of quantitative Western blots. The expression levels for the two strains were comparable and in many conditions, within the accuracy of our technique (see below *MscL Channel Counts with Quantitative Western Blots* for details).

To establish the gating functionality of our fusion, we integrated the coding region of MscL-sfGFP into the osmotically susceptible MJF612 strain (a generous gift of Ian Booth), which had four mechanosensitive channel genes (*ΔmscL, ΔmscS, ΔmscK*, and *ΔybdG*) [39] knocked out. We performed a series of osmotic shock survivability assays (see *Plaque/Survivability Assays* in Supporting Information S1 for details). We observed a considerably higher survival rate (Figure S1) for cells expressing our fusion (71±13% for 0.5 M NaCl osmotic shock) as compared to the rate from the MJF612 strain (15% for 0.5 M NaCl osmotic shock), but a lower survival rate as compared to that from the WT strain MG1655 (defined as 100%). For comparison, the work of Levina *et. al* reported a survival rate difference of ∼10% between a similar WT (Frag1) and mutant strain (MJF429) [19]. Part of this discrepancy may be explained by the exact choice of protocol. We have observed that survival rates can be systematically influenced by factors other than the functionality of the channel, for example, speed of mixing and choice of media. To directly measure channel activity, it has been shown that spheroplasts made from the MLG910 strain demonstrate electrophysiological activity of MscL-sfGFP which is nearly identical to that of WT MscL (unpublished data, A. Rasmussen and I.R. Booth). Taken together, these results suggest the MscL-sfGFP fusion is functionally similar to WT MscL. We also used fluorescence microscopy to check for the proper insertion of MscL in the cell membrane. For a limited set of data, we used confocal microscopy and fluorescence recovery after photobleaching (FRAP) [40] to determine that the majority of the fusion proteins are mobile in the membrane (Figure S2). We interpret all these findings as an indication that the MLG910 fusion strain is a fair representation of the wild-type MG1655 strain.

### MscL Channel Count with Quantitative Western Blots

To establish the impact of stress factors on the expression levels of MscL, we used quantitative Western blots. We prepared lysates derived from the various strains, grown in three different media to early exponential (OD_600_ 0.3) and stationary phase (OD_600_ 1.2–1.7). Known volumes of lysates were run alongside purified protein references (either MscL or MscL-sfGFP) of known concentration. The references were diluted in MJF612 lysate to keep the total non-specific protein loaded similar to the whole cell lysates. Reference proteins and lysates were separated by SDS PAGE, transferred to nitrocellulose membranes, and immuno-stained with primary antibodies for either MscL or GFP (as discussed below). Detection of the bands was achieved by imaging the chemiluminescence resulting from horseradish peroxidase (HRP) labeled secondary antibodies. By measuring the relative intensity of the bands and comparing them to the purified protein reference bands, we determined the mean number of channels per cell for a given condition (see *Western Blots* in Materials and Methods for details).

**Figure 1:**
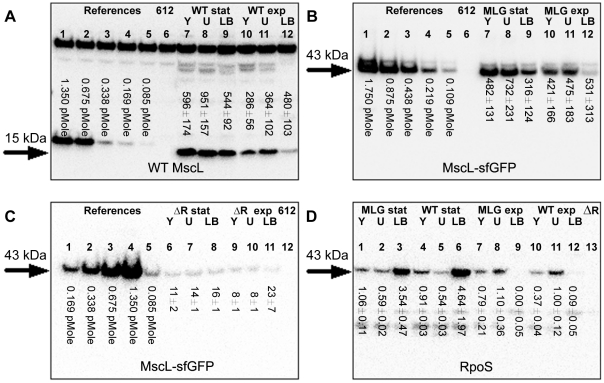
Representative Western blots showing expression of MscL, MscL-sfGFP, and RpoS. Arrows indicate the protein of interest. Other bands are the result of non-specific binding. The strains of interest were cultured to exponential (exp) and stationary (stat) phase in LB-Miller (LB), M9+glucose (U), and M9+glycerol (Y). In Figures 1A, 1B, and 1C lanes 1 through 5 are a concentration series of a known number of purified channels diluted into lysate from the MJF612 strain (References). The numbers under lanes 6 through 12 represent the average number of channels from three independent Western blots for the respective conditions. The total error of each measurement includes contributions from the standard deviation of 3 repetitions and the systematic uncertainties in the absolute calibration related to chemiluminescence linearity, initial cell culture density, and lysis efficiency. (A) Western blot performed with MscL antibodies. Lysate from the MJF612 strain (612) was used as a negative control (lane 6). Lanes 7 through 12 show the MscL levels in the MG1655 strain (WT). (B) Western blot performed with GFP antibodies. Lysate from the MJF612 strain (612) was used as a negative control (lane 6). Lanes 7 through 12 show the MscL-sfGFP levels in the MLG910 strain (MLG). (C) Western blot performed with GFP antibodies. Lysate from MJF612 strain (612) was used as a negative control (lane 12). Lanes 6 to 11 show the MscL-sfGFP levels in the MLG910-Δ*rpoS* strain (ΔR). (D) Western blot performed with RpoS antibodies. Lysate from the MLG910-Δ*rpoS* strain (ΔR) was used as a negative control (lane 13). Lanes 1 to 12 show the RpoS levels in the MLG910 (MLG) and MG1655 (WT) strains. The numbers under the lanes are the relative amount of RpoS, as compared to the lysate from M9+glucose (lane 11), determined by the average of three independent repetitions. The errors are the standard deviation.

For the MG1665 strains, we used a polyclonal antibody for MscL (a generous gift of S. Sukharev), which showed multiple bands in every lane (Figure 1A), presumably due to the polyclonal nature of the antibody. We were able to identify the specifically labeled MscL bands (15 kDa) from the nonspecifically labeled bands (see Western Blots in *Materials and Methods* for details). The MscL-sfGFP fusion protein is ∼43 kDa, which overlaps with some of the nonspecific bands of the MscL antibody. Accordingly, to accurately measure the expression of MscL-sfGFP in the MLG910 strain, we used a monoclonal GFP antibody which displayed higher specificity towards the MscL-sfGFP fusion protein (Figure 1B). As an additional consistency check, we measured the expression levels of MscL-sfGFP in the MLG910 strains using the MscL antibody and found comparable results (see Figure S3 for details).

The Western blot measurements showed much higher MscL expression levels, as compared to previously published results (Table 1). In LB-Miller medium, we did not observe a resolvable change in MscL levels with increasing age of the culture (OD_600_) (lanes 9 and 12 of Figures 1A and 1B). However, in the slower growth-rate M9-based media, the number of MscL channels per cell increased by nearly 2-to-3 fold during the transition period from exponential to stationary growth phase (lanes 7 and 8 vs. 10 and 11 in Figures 1A and 1B), similar to previous findings [16]. To slow the growth rate and increase the potential stress created by our M9 media, we omitted optional supplements (thiamine, Casamino acids, and riboflavin) typically used to increase the growth rate. In addition, we used two different carbon sources, glucose and glycerol, where the nutritionally poorer glycerol reduced the growth rate by a factor of two or more, depending on the culture conditions. These results demonstrate that the carbon source and associated growth rate influence the amount of upregulation of MscL during the transition from exponential to stationary growth phase.

When cells are in late exponential phase or subjected to environmental stresses, an alternative sigma factor known as RpoS is activated [26]. Earlier work has examined how the expression of mechanosensitive channels is affected by RpoS expression [16] and we have explored this as well. We measured the level of MscL-sfGFP protein in the MLG910 strain where the *rpoS* gene had been knocked out (MLG910-Δ*rpoS*). As expected [16], the expression of MscL-sfGFP protein in the MLG910-Δ*rpoS* strain was significantly lower (Figure 1C, lanes 6-11). In contrast to the previous results for the MG1655 and MLG910 strains, we observed neither an increase in channel expression upon entry into stationary phase nor a change in expression level due to different carbon sources, as previously noted [16]. We take these results to suggest that MscL expression in the Δ*rpoS* strain represents the baseline level of MscL expression in the absence of any stress.

Next, we performed a relative-comparison Western blot for the MG1655 and MLG910 strains with antibodies for RpoS (Figure 1D), where all lanes were normalized to lane 11 (the MG1655 strain grown in M9+glucose media). To ensure consistency, the RpoS blot used aliquots from the same lysates used in Figures 1A and 1B. In LB-Miller media we observed that RpoS levels were nearly absent or relatively low for cells in exponential phase (Figure 1D, lanes 9 and 12) as compared to those found in stationary phase (Figure 1D, lanes 3 and 6), as previously reported [28]. For cells grown to exponential phase in M9+glucose and M9+glycerol, there were noticeable levels of RpoS present (Figure 1D, lanes 7, 8, 10 and 11), consistent with previous results [28], [29], [30]. In this context, we can interpret the up-regulation of MscL, at least in M9 media, as a response to carbon source related stress. In stationary phase, we observed media-dependent changes in RpoS expression levels. For cultures grown in LB-Miller, there was a dramatic 40-fold or greater upregulation of RpoS levels (Figure 1D, lanes 3 and 6 vs. 9 and 12). For three conditions in minimal media (Figure 1D, lanes 1, 2, and 5 vs. lanes 7, 8, and 11), the measured levels of Rpos showed more modest changes, decreasing by 1.2–1.9 fold. The remaining condition in minimal media, MG1655 grown in M9+glycerol, showed an inconsistent trend (Fig. 1D lanes 4 vs. 10) of increasing RpoS levels. We note that interpretation of these results can be complicated by native proteolysis of RpoS, which depends on the growth media and growth phase, leading to substantial variability of the RpoS protein half-life ranging from 1 minute to over 30 minutes [28], [29], [30], [41].

### MscL Channel Count with Fluorescence Microscopy

**Figure 2:**
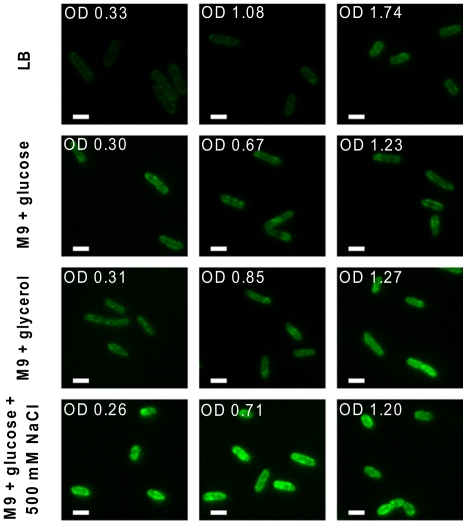
Typical fluorescence microscopy images of MLG910 for various conditions. The white scale bar is 2 µm long. The relative contrast of the individual images has been unaltered. The contrast of the overall composite image has been adjusted for clarity.

To conduct a single-cell based census of MscL channels, we used epi-fluorescence excitation microscopy to image the MLG910 strain under various growth conditions and stages. We imaged multiple fields of view, typically analyzing >1000 immobilized bacterial cells per condition. Under most conditions (except where noted), we observed a dynamic, somewhat grainy distribution of fluorescence primarily localized along the entire cell perimeter with no strong preference for the poles of the cell (see Figure 2 for representative images), which appears to be in contrast to previous findings [42]. Occasionally, we observe there are mobile puncta (1∼3 per cell), where each punctum contains less than 5% of the total integrated fluorescence from the cell. These puncta do not show a preference for the cell poles. However, for cells grown to stationary phase or in media supplemented with excess salt (500 mM NaCl), less than 5% of the cells exhibit static, nearly diffraction-limited punctate features, typically found at one pole. The total integrated number of fluorescence counts of each cell was determined and converted into the total number of fully assembled channels by using a calibration factor of fluorescence-counts-per-sfGFP (see *Single Molecule Fluorescence Calibration* in Materials and Methods for details), explicitly assuming the fluorescence of each sfGFP represents a single channel subunit and that five subunits form a fully assembled channel [12], [43].

**Figure 3:**
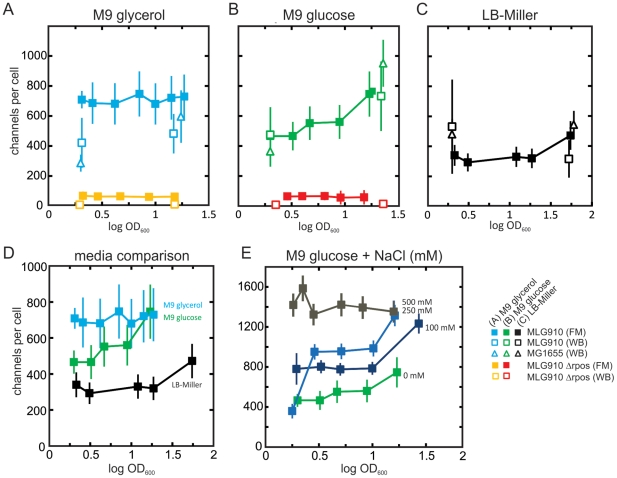
Mean channel counts per cell determined by fluorescence microscopy (FM) for various media versus OD_600_. (A) M9+glycerol. Fusion strains with (MLG910, light blue squares) and without RpoS (MLG-Δ*rpoS*, yellow squares). (B) M9+glucose. Fusion strains with (MLG910, green squares) and without RpoS (MLG-Δ*rpoS*, red squares). (C) LB-Miller. Fusion strains with RpoS (MLG910, black squares). In Figures 3A, 3B, and 3C, the corresponding mean number of channels determined by Western blots (WB) for the MLG910 strain (open squares) and the MG1655 strain (open triangles) are shown for reference. (D) Comparison of fluorescence microscopy results from MLG910 grown in three different media. (E) Comparison of fluorescence microscopy results from MLG910 grown in M9+glucose supplemented with four different NaCl concentrations: 0 mM (green squares), 100 mM (dark blue squares), 250 mM (gray-blue squares), and 500 mM (dark gray squares). The error bar of each fluorescence microscopy results measurement is dominated by systematic uncertainties in the absolute calibration related to single-molecule fluorescence calibration. The standard error of the mean of the uncalibrated fluorescence counts per cell is typically less than 5% of the total error bar.

In agreement with the quantitative Western blots, these measurements indicated that the media and the age of the culture affected the expression level of MscL-sfGFP (Figure 3). The fluorescence microscopy measurements indicated that the mean number of channels per cell determined by total integrated fluorescence is on the order of 300–1400 (10^2^ to 10^3^ channels per µm^2^), depending on culturing conditions.

The number of channels increased in M9 minimal media as compared to cells grown in LB-Miller media (Figures 3A and 3B compared to 3C). Depending on the carbon source, there was a 2.5-fold (M9+glycerol) or a 1.5-fold (M9+glucose) increase in the number of MscL-sfGFP channels per cell in the early exponential phase of growth. Interestingly, as the M9-media-grown cells entered stationary phase, they reached similar expression levels for both carbon sources (Figure 3D).

We also observed that the number of channels per cell increased steadily with salt concentration (Figure 3E), presumably demonstrating osmotic induction [16] related to the increased osmolality (mOsm/kg) of the media (Table S1). During exponential phase, cells grown in the various salt-supplemented M9 media - M9+glucose+100 mM NaCl (342 m Osm/kg), M9+glucose+250 mM NaCl (529 mOsm/kg) and M9+glucose+500 mM NaCl (886 mOsm/kg) - showed a 2–3 fold increase in protein expression, as compared to cells grown in M9 media without supplemented salt (234 mOsm/kg). Cells grown to stationary phase in the various salt-supplemented media appear to be approaching a common expression level of ∼1300 channels per cell, nearly twice the maximum level seen in M9 media without salt (∼700 channels per cell).

The age of the culture (OD_600_) also influenced the mean number of MscL proteins per cell. In the presence of 100 mM or 250 mM NaCl salt, the cells showed a characteristic increase in expression level around OD_600_≈1, which can be interpreted as arising from the stress associated with transition to stationary phase. However, cells grown in the presence of 500 mM NaCl did not show this tendency. Instead, we observed a fairly-constant, relatively-high level of expression, which may represent a maximum level of MscL expression in response to salt and growth.

To summarize, the results from both experimental methods showed a general trend of increasing MscL levels as the quality of the media's carbon source was decreased, in going from LB-Miller to M9+glycerol, and as the cultures entered stationary phase. The agreement between the mean values found from fluorescence microscopy and quantitative Western blots is summarized in Table 2.

**Table 2:** Comparison of channel counts per cell from quantitative Western blots and fluorescent microscopy.

Condition			Western blot		Fluorescence Microscopy	
Strain	Media	Growth Phase	OD_600_	Mean number	OD_600_	Mean number
MG1655	LB-Miller	Exponential	0.3	480±103^a^	-	-
MG1655	M9+glucose	Exponential	0.3	364±102^a^	-	-
MG1655	M9+glycerol	Exponential	0.3	286±56^a^	-	-
MLG910	LB-Miller	Exponential	0.3	531±313^b^	0.33	340±68
MLG910	M9+glucose	Exponential	0.3	475±183^b^	0.3	466±64
MLG910	M9+glycerol	Exponential	0.31	421±166^b^	0.31	709±57
MG1655	LB-Miller	Stationary	1.78	544±92^a^	-	-
MG1655	M9+glucose	Stationary	1.36	951±157^a^	-	-
MG1655	M9+glycerol	Stationary	1.24	596±174^a^	-	-
MLG910	LB-Miller	Stationary	1.72	316±124^b^	1.74	472±95
MLG910	M9+glucose	Stationary	1.34	732±231^b^	1.23	746±150
MLG910	M9+glycerol	Stationary	1.17	482±131^b^	1.27	729±147

a Western blots performed with MscL antibody.

b Western blots performed with GFP antibody.

MG1655 and MLG910 were cultured in LB-Miller media (LB), M9 minimal media supplemented with glucose (U), and M9 minimal media supplemented with glycerol (Y) to the indicated optical density (OD600).

### Distribution of MscL Subunits in a Population

**Figure 4:**
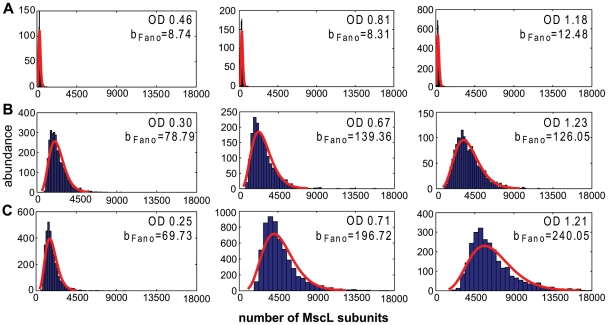
Distribution of MscL Subunits under Different Growth Conditions at Various Stages of Growth. The red curves show the fitting of the gamma distribution to the histograms. The OD_600_ and the Fano factor b_Fano_ for a given sample are listed. (A) MLG910-Δ*rpoS* strain grown in M9+glucose to OD 0.46, 0.81, and 1.18, respectively. (B) MLG910 strain grown in M9+glucose to OD 0.3, 0.67, and 1.23, respectively. (C) MLG910 strain grown in M9+glucose+0.5 M NaCl to OD 0.26, 0.71, and 1.2, respectively.

In addition to measuring the mean number of fully-assembled channels, N_mean_, we used the fluorescence dataset to determine the population distribution of MscL monomer subunits expressed under various growth and stress conditions (see *Single Cell Fluorescence Microscopy* in Materials and Methods for details). Not only are such measurements a first step towards addressing the important question of the cell-to-cell variability of these channels in living bacteria, but they also provide insights into the largely unexplored nature of the regulation of these channels under different physiological conditions. Under different conditions, we observed changes in the width and shape of the respective protein distributions (Figure 4). Though earlier work has given snapshots of the expression level distributions of other proteins in cells [5], [6], [8], the kind of systematic analysis as a function of both growth media and OD done here provides further clues as to additional physiological roles that these channels might play as well as into the detailed nature of their regulation which until now remains completely unclear. To characterize the distribution widths, we determined the standard deviation, σ, and Fano factor (b_Fano_) for each distribution, where b_Fano_ is given by
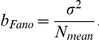



The Fano factor is a measure of the non-Poissonian character of the distribution, where a Poisson distributed protein abundance would correspond to b_Fano_ = 1. The MLG910-Δ*rpoS* strain did not show a wide variation in the Fano factor value with increasing OD_600_ (Figure 4A and Table S2). We interpret these Fano factor values as the baseline level of population variability in the absence of carbon source or salt associated stress. There was a dramatic increase in the Fano factor of the RpoS-expressing MLG910 strain distributions, as compared to the MLG910-Δ*rpoS* strain (Figure 4B and Table S2).

The presence of RpoS alone caused a 10-fold increase in the Fano factor value. In the presence of 250 mM NaCl, an even larger 10–30 fold increase was observed (Figure 4C and Table S2). This increase depended on the OD_600_ of the culture, indicating that the presence of salt introduced further changes in the expression profile. The results for all the tested conditions are summarized in Table S2.

Numerous theoretical models have linked the steady-state, gene-expression distribution of a population to stochastic factors describing the transcriptional and translational processes of a single cell. One of the simplest descriptions results in a steady-state distribution described by a gamma distribution of the form
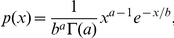
where *p(x)* is the probability of occurrence of x protein subunits, *a* is a measure of the rate of transcriptional bursts, and *b* is the measure of size of the corresponding translational burst [35]. For this class of models b = b_Fano_. We fit this distribution to our data as a crude gauge of the complexity of MscL regulation. For the cells expressing RpoS, we observed an increasing trend of both the *a* and *b* parameters with growth phase, and/or salt levels (Table S2). In general, the distributions were reasonably described by such a fit in early exponential phase, but outside this phase or in the presence of salt, the gamma distribution did not account well for our data (last two panels of Figure 4C).

## Discussion

### Two Independent Methods Lead to Higher Than Expected MscL Expression Levels

In this work, we present a robust approach to counting the absolute number of MscL channels and studying the changes in its expression caused by various stress factors. The strategy is general enough to be applied to other membrane proteins. We use two independent methods: quantitative Western blots for bulk measurement and fluorescence microscopy for measurement at the single cell level. Both methods obtained comparable results, showing somewhat similar numbers of channels and the same general expression trends with media choice and growth phase (Table 2). Together, these techniques provide a much more convincing picture. As an additional consistency check, we note that our relative expression level changes associated with carbon source, growth phase and osmolality for each method are of a similar scale to those previously reported [16]. These observations suggest that the absolute numbers of MscL channels measured in our study are a representative *in vivo* census, with certain exceptions detailed below.

Both methods have their associated systematic uncertainties. For example, quantitative Western blots may be affected by incomplete protein extraction or proteolysis that may occur during lysate preparation (see *Lysate Preparation* in Materials and Methods for details). In the case of fluorescence microscopy, photobleaching, misfolding [44] , lack of maturation [45], or self-quenching of sfGFP molecules will lead to a systematic reduction of total fluorescence. For example, homo-polar excitation transfer (self-quenching) may be expected when sfGFP molecules are roughly within one Forster radius, as may be the case when MscL-sfGFP is in pentameric form or when clusters are formed. Based on our observations (see *MscL Channel Count with Fluorescence Microscopy* in Results), we expect most of the channels will not be in clusters. If self quenching occurs, the effect will primarily affect the pentameric form of MscL-sfGFP. The total fluorescence of a pentamer will be less than the fluorescence of five well separated monomers. With these considerations in mind, even after controlling for many of these factors, each method likely results in an undercount. However, our results still indicate that the total absolute number of MscL proteins per cell is at least an order of magnitude higher than previously reported numbers (Table 1).

### Induction of MscL Expression Is Not Described Well by a Simple Biophysical Model

To our knowledge, we are the first to measure the cell-to-cell variability of MscL expression, which varies substantially with culturing conditions. In this work we describe these changes quantitatively by determining the Fano factor empirically from the distributions and by fitting a gamma distribution to the histograms of MscL monomers at the single cell level (red curves of Figure 4 and parameters listed in Table S2). The gamma distribution is derived from a model which assumes: the mRNA expression level is determined by a single-state, unregulated promoter; the proteins are expressed in translational bursts from a single copy of mRNA; the number of proteins per burst event is described by an exponential distribution; and the translation events are uncorrelated in time. There is no transcriptional “feedback” such as auto-regulation or regulation by another gene. The rate of translation is a constant. This distribution accurately describes the measured expression levels of a few simple model genetic circuits in *E. coli* [46], [47]. For low stress conditions (exponential phase and/or media without supplemented salt), the gamma distribution represent the data well. In stationary phase, when significant regulation by RpoS occurs, or in the presence of salt, when osmotic induction occurs, the gamma distribution does not provide a good fit for our data. This suggests that a simple single-promoter state model of transcription [35] may not be adequate to quantitatively describe the observed increase in MscL expression under these conditions. We must bear in mind that the conditions leading to the derivation of model distributions make specific assumptions about both transcription and translation that may not be satisfied in the context of these genes. For example, distributions more complicated than the gamma distribution can arise when there is a multiple-state promoter or multiple promoters involved [48]. Further, the regulatory behavior of RpoS introduces another layer of complexity not considered in these models. It may not even be possible to distinguish between different biophysical models solely on the basis of fitting calculated distributions to measured steady-state protein distributions [49]. As a result, it is really only as a point of departure that we make preliminary fits to our data in terms of gamma distributions, knowing full well that the situation can be (and is) more complicated. In many ways, our thinking is analogous to that used when Hill functions are used to fit binding data since such fits can provide a convenient summary of large quantities of data [50] without necessarily reflecting any underlying mechanistic picture of the binding process.

### Osmotic and Carbon Source Induction Mechanisms Produce Comparable MscL Expression Levels

The results summarized in Figure 3E reconfirm earlier findings that demonstrate MscL levels can be raised by increasing the salt of a given culture media [16]. One explanation of this trend is that the cells are responding to the increased osmolality of the media and preparing for a possible hypo-osmotic shock. For comparison, it is interesting to consider the amount of induction caused by a poor carbon source (Figure 3A vs. 3E). For example, the levels of MscL present in the MLG910 strain grown in M9+glycerol (277 mOsm/kg) are comparable to those found in M9+glucose+100 mM NaCl (342 mOsm/kg), where the growth rates in both media are similar, and within ∼20% of those found in M9+glucose+250 mM NaCl (529 mOsm/kg). This would imply that carbon source induction mechanisms are, at least, on a comparable scale to salt induction related ones. It is unclear if the response to carbon source related stress has a specific physiological purpose or if it is a passive response. In previous work [51], it was found that some of the genes that were up-regulated during starvation conditions were associated with metabolism and transport. However, the same study also noted many of the upregulated genes were not associated to any specific response.

### RpoS Expression Level is Not the Only Factor Impacting MscL Expression

We find that the presence of RpoS leads to much higher channel counts in RpoS expressing strains than in the MLG-Δ*rpoS* mutant (Figs. 3A, 3B, and 4A vs. 4B) thus confirming RpoS plays an important role in MscL regulation [16]. The effect that a given level of RpoS protein has on regulation depends on how many of the RpoS subunits assemble into full RNAP-RpoS holoenzymes. The RNAP-RpoS regulatory network is governed by multiple factors which lead to a complex, shifting balance between production, inhibition, and proteolysis [28], [52], [53]. Thus, it can be difficult to establish a clear quantitative relationship between RpoS and MscL levels. For example, cells grown to stationary phase in LB-Miller showed the highest levels of RpoS by far (4-fold or higher), although the levels of MscL were roughly half of those found in cells grown to stationary phase in minimal media. In this case, it is possible that a sizeable fraction of the RpoS subunits are prevented from forming RNAP-RpoS holoenzymes by anti-sigma factors and/or other proteins that act like anti-sigma factors, such as RssB [41], [54], [55], [56]. While it is also possible that the accuracy of our RpoS measurements is affected by the considerable variation in turnover for RpoS [28], [29], [30], it does appear, nonetheless, there are factors other than the absolute level of RpoS required to explain the observed trends. For example, we note that even without RpoS, there are other means of MscL induction. We observed the salt dependent upregulation of channels in the MLG910-Δ*rpoS* strain (Figure S4), as has been previously noted [16].

### Implications for Electrophysiology

There is a considerable discrepancy between our measured total number of channels and the reported number of active channels from the work summarized in Table 1. One possibility is that experimental uncertainties and variations in cell culture conditions may have created this artificial divide. Another distinct possibility is that the vast number of channels we observe are inactive. From our FRAP studies (Figure S2) and Western blots performed on the cytoplasmic fraction of cell lysate, we estimate less than 10% of the total observed MscL is found in the cytoplasm and the remainder should be in the cell membrane. If the inactive fraction of proteins occurs *in vivo*, it may be caused by improper assembly (e.g. incorrect stoichiometry or misfolding), improper insertion, or inactivation (i.e., by tension). On the other hand, it is also possible that proteins are inactivated during *in vitro* sample preparation prior to electrophysiological measurements, notably the requirement to produce large spheroplasts amenable to patch clamp experiments. Finally, since MscL channels gate at tensions near the point of membrane rupture, an underestimation of the total number of channels can occur if the patch breaks before reaching saturating tensions. A possible test of these ideas would be to apply the quantitative fluorescence techniques demonstrated in this current work to an electrophysiological setting. The total number of fluorescently tagged proteins within a patch or spheroplast could be measured and compared with the number of electrophysiologically-active channels.

### A Multitude of MscL Channels Can Impact Gating Activity

At this time, it is unclear what fraction of channels we observe *in vivo* is active. Given the relatively large number of channels we observe (N_total_≈300–1000), it is interesting to speculate on how these numbers might impact channel gating behavior, if we assume all the channels are active.

For example, channel activity *in vivo* may occur at lower tensions than might be expected. As a simple illustration of this effect, we calculate the average number of open channels, *N_open_*, resulting in
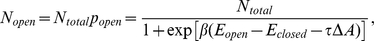
where *p_open_* is the probability of a channel being open, τ is the applied tension, *ΔA* is the area difference between open and closed state, and *E_open_* and *E_closed_* are the energies of the open and closed state, respectively [50]. From this expression, we find the critical tension, *τ_c_*, required to open one channel on average (i.e. when *N_open_* = 1) is 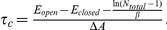


**Figure 5:**
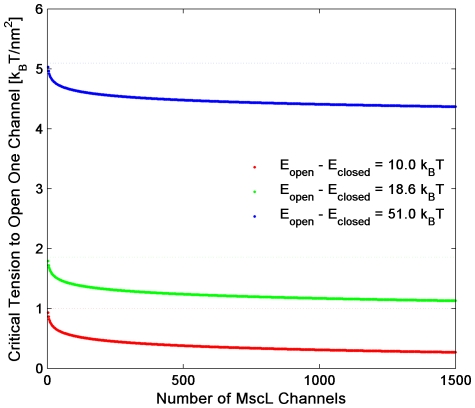
The dependence of critical tension needed to open one channel on the total number of channels. Estimates of E_open_-E_closed_ show considerable variation due to experimental uncertainties and the effect of lipid composition [15]. For illustration, we chose representative values found in the literature that reflect the range of values: 10.0 k_B_T (red solid line) [62], 18.6 k_B_T (green solid line) [63], and 51.0 k_B_T (blue solid line) [64]. Dashed lines show τ_1/2_ the tension, where P_open_ = 0.5. The area change ΔA was taken to be 10 nm^2^ .

What this expression shows is that by having multiple channels present, the tension needed to open at least one channel is reduced (Figure 5). There is an effective lowering of the gating energy (*E_open_ - E_closed_*) by ln(N_total_)/β = 5∼7 k_B_T. The tension requirement for gating one channel (or very few) can be considerably lower than τ_1/2_, the tension where P_open_ = 0.5 (dashed lines of Figure 5). It is commonly noted that the value of τ_1/2_, determined from electrophysiological measurements, is very close to the tension when a typical membrane ruptures in the range between 1 and 3 k_B_T/nm^2^. By having several hundred channels, a bacterium may only need to activate a small percentage of channels, rather than half, thus operating in tension ranges below lytic values.

Also, recent work has predicted that at sufficiently high protein areal-densities, channels can demonstrate cooperative gating [22]. In the range below ∼10^−3^ channels per µm^2^, channels essentially gate independently of each other. However, for our measured values, 10^2^–10^3^ channels per µm^2^, the probability of two channels opening at-a-time can be equal to or even greater than the probability of one channel opening as the applied tension is increased to values approaching τ_1/2_.

### Relevancy to Osmotic Protection and Survivability

Currently, there are two analytically modeled mechanisms for mechanosensitive channels to protect the cell membrane from rupture: transport of water and/or osmolytes which is mediated by conductance through open channels [20], [21] and expansion of non-conductive MscL proteins [57] which adds extra area to the membrane. Both types of models predict greater protection with greater numbers of channels.

As a numerical example, previous work [21] has estimated that the membrane tension experienced by a bacterium-sized vesicle under a potentially-lethal osmotic shock (∼300 mOsm) can be effectively reduced below lytic levels if the number of activatable MscL proteins exceeds ∼100. We are, for the moment, neglecting the contributions of the other mechanosensitive channels found in wild type cells. Based on this model, with our typical mean numbers, osmotic protection can be extended to shocks of ∼500 mOsm, in reasonable agreement with the observed hypo-osmotic challenge assays [19]. Interestingly, in experiments comparing the survival rates of the wild type strain MG1655 (∼300 MscL channels) and the mutant strain MLG910-Δ*rpoS* (10–20 MscL channels), we observe no significant difference in survival rates for shocks up to 500 mOsm (Figure S5), similar to previous results [16]. However, a more complete analysis requires consideration of the other mechanosensitive channels.

Since MscL has the highest gating tension (neglecting the effects discussed in previous section), the other channels will likely be activated before MscL in a hierarchical fashion, according to their respective gating tensions. Also, it is known that the presence of MscK or MscS, by themselves at physiological expression levels, is sufficient to provide some protection or even complete rescue from downshock [19], respectively. The relative number of MscS channels has been reported to be 2–5 times greater than the number of MscL channels, even for *rpoS* deletion strains [16]. In this context, it is likely we are overestimating the impact of MscL levels on osmotic rescue for wild-type like strains. In order to include these effects, we need to know the water conductance and the absolute numbers of each of the other channels. At this time, these data are less certain than the data for MscL. This motivates the need for future censuses for the other mechanosensitive channels.

The techniques demonstrated in our current work may provide another means of addressing the connection between survivability and channel expression level. For example, we could image our calibrated fluorescent strains during osmotic shock and throughout the recovery process. In principle, it should be possible to determine how much enhanced MscL levels in wild-type and various channel deletion strains affect survivability with single-cell resolution. If it is true that more channels leads to higher survival rates, it is interesting to speculate on how the variability of the cell-to-cell MscL distributions (as measured by the Fano factor) increases substantially with stress, age and salt (Figs. 4B and 4C). It is possible that the increased variability is a population survival strategy. The majority of cells would not be required to prepare for a worst-case possible osmotic catastrophe. A population of cells can “hedge its bets” on a small percentage of cells in the highest expressing region of the distribution. If survival rates turn out to be weakly dependent on the number of channels, it begs the question of why is there an apparent excess of channels and why is there upregulation of these channels under stress conditions?

## Materials and Methods

### Chromosomal Integration

A construct that coded for a MscL-sfGFP fusion protein (see Figure S6 and *Fusion Protein Design and Sequence* in Supporting Information S1 for details) was chromosomally integrated in to the MG1655 strain by recombineering with lambda Red-mediated homologous recombination [58]. We chose a negative selection scheme to avoid introducing an antibiotic resistance marker in the fusion construct (see Figure S7 and *Chromosomal Integration Strategy* in Supporting Information S1 for details). To verify successful integration, multiple colonies were picked for single colony PCR amplification of the MscL region. The mass of the desired fragment was confirmed by agarose gel electrophoresis. The PCR fragments were purified and sent for DNA sequencing (Laragen) for final verification.

### Growth Conditions and Cell Preparation

Starter cultures were grown aerobically in 5 mL of LB-Miller media [31] in the presence of appropriate antibiotic (except MG1655 strain) at 37°C overnight. The following morning, LB-Miller or M9 minimal media (without supplements) was inoculated at 1∶1000. The carbon source in minimal media was 0.5% glucose or 0.5% glycerol. Samples for Western blots (∼900 mL) were grown in 2L flasks to the desired OD_600_. The volume of the culture was measured and cells were pelleted by centrifugation (Beckman-Coulter Avanti J-20, 6000 rpm for 15 min at 4°C). Pellets were frozen overnight (−20°C) and resuspended in 50% glycerol to a volume of 10 mL. Samples for microscopy were grown as described earlier in 50 mL of desired media. Five 1 mL aliquots at different values of OD_600_ were used to prepare the sample used for fluorescent microscopy data collection.

### Lysate Preparation

Each cell resuspension (for Western blots) was diluted in TBS+SDS buffer (20 mM Tris, 150 mM NaCl, 1% SDS wt/wt) to a concentration equivalent to cell culture of an OD_600_ of ∼10. 45 ml of this mixture was taken and mixed ∼10 times with a serological pipette, until a large change in turbidity was observed. 350 µL of PMSF (phenylmethylsulfonyl fluoride) protease inhibitor (Sigma P7626) were added to each lysate, followed by additional pipette mixing. Next, each sample was homogenized using a blade-based homogenizer for 15–30 s to shred the remaining DNA and insoluble fraction. At this level of preparation, samples could be run on gels. However, upon ultracentrifugation (50,000 g), these samples typically still produced a pellet, indicating the presence of murein sacculi or possibly even unlysed cells. More importantly, when these lysates were run on a gel, on occasion, we observed poor band formation, streaking or clogging of lanes. To ensure complete lysis and consistency of successive gel runs, the samples were clarified by passing them 5 times through a microfluidizer (Microfluidics M-110L) operating at 4°C and ∼18000psi. Approximately 10 to 15 mL of the undiluted fraction was collected for Western blot analysis. These samples produced no measurable pellet after ultracentrifugation and ran consistently on gels over multiple repetitions. The addition of the homogenizer and microfluidizer steps led to a marginal gain, typically 5%–10%, in the measured number of detected MscL channels. Any error due to concentration changes or proteolysis introduced by these additional stages was estimated by a mock lysis procedure see Figure S8 and *Protein Loss During Lysate Preparation* in Supporting Information S1 for details) and is reflected in the error bars for the Western blot measurements.

### Western Blots

SDS-PAGE was performed with fixed percentage crosslinked gels (NuPAGE Novex 8% Bis-Tris, Invitrogen WG1002BX10) to ensure even transfer of the proteins across the entire running length of the gel. In each lane, we loaded a predetermined volume of each corresponding lysate, mixed with NuPAGE LDS Sample buffer (Invitrogen NP0007). The volume of lysate was adjusted to provide a load of ∼20 µg of total mass of cells (∼20 µl of lysate at OD_600_ ∼10) for each lane. We empirically determined that this is the maximum total mass of cells that can be loaded per lane which still leads to consistent blotting results. As a negative control, we used the lysate from the MJF612 strain. Serial dilutions of purified protein (generous gift from Troy Walton) were run alongside with the lysate and were used for calibration. Loaded gels were run in MES buffer (Invitrogen NP0002) at 200 V for 35 minutes. The proteins were transferred using Nitrocellulose iBlot Gel Transfer Stacks (Invitrogen IB3010-01) and blocked in TBS 5% milk 2% BSA buffer (20 mM Tris, 150 mM NaCl, pH 7.5) for 1 hour at room temperature. The primary antibody recognizing the appropriate epitope – MscL (gift from S. Sukharev), GFP (Roche Applied Science 11814460001) or RpoS (Santa Cruz Biotechnology sc-101602) - was added to the blocking buffer at a dilution of 1∶5000 for overnight incubation at 4°C. The next day, membranes were washed twice with TBS 5% milk 2% BSA buffer and incubated 1 hour at room temperature in the appropriate HRP-conjugated secondary antibody - anti-rabbit (GE Healthcare NA934VS) or anti-mouse (GE Healthcare NA931VS). After incubation, the membranes were subjected to three successive 5 minute washes in TBS buffer and developed for 5 minutes in SuperSignal West Femto Maximum Sensitivity Substrate (Thermo Scientific 34095). Developed membranes were imaged with a home-made gel imager that used an EMCCD chip camera (Andor DU-897E). Typical exposure times were 100 ms to 1 s.

Over the course of a few Western blots, the reference protein concentrations were empirically adjusted to encompass the range of chemiluminescence shown from the bands of interest. For each quantitative Western blot, we measured the intensities of the reference bands and the lysate bands (Figure S9A, first row) and normalized them to the dimmest band. From the reference band intensities and knowledge of how many channels we loaded into the references (Figure S9A, second row), we established a linearly-fit, calibration curve of relative intensity vs. number of channels (Figure S9B). From the calibration curve, we extrapolated the total number of channels present in each lysate band based on its intensity (Figure 9A, third row). We found the equivalent number of cells loaded in each lane (Figure S9A, fourth row) by calibrating the cell density of the original cell culture using disposable hemocytometers (see Table S3 and *Calibrating the Cell Density for OD_600_ Measurements* in Supporting Information S1). Typically, the actual equivalent number of cells loaded in each lane ranged from 0.4×10^8^ to 3×10^8^. From these data, we determined the number of channels per cell (Figure S9A, fifth row).

The anti-MscL blots showed multiple bands for a given lane (Figure 1A), presumably related to the polyclonal nature of the antibody. We identified the legitimate MscL bands (15 kDa) by observing which of the bands from the reference protein concentrations (Figure 1A, lanes 1 through 5) demonstrated an intensity trend that matched the concentration trend of the reference proteins (progressively increasing by a factor of two). For the lanes containing cell lysates, we identified the MscL bands by matching their running positions on the blot to the reference MscL bands (15 kDa marker on Figure 1A), allowing us to discriminate against the non-specific bands (25 kDa, 40 kDa, ∼75 kDa). The control MJF612 lysate, which has MscL deleted, establishes there is no detectable non-specific band in the mass range near 15 kDa. For the anti-GFP blots, we note that, within our detection limit (5∼10 molecules/cell), there are no bands corresponding to free sfGFP (∼23 kDa), indicating the vast majority of sfGFP molecules are attached to MscL.

### Single Cell Fluorescence Microscopy

A ∼3 µL sample of the culture was placed between two RCA-SC2 cleaned [59] low-autofluorescence glass (Corning D-263) cover slips. The edges of the slips were sealed with VALAP, forming a 22 µm×22 µm×2 µm viewing chamber. The cells were imaged in laser-excited (473 nm) epi-fluorescence mode at 100× magnification (Olympus NA 1.45 TIRFM objective) using a homemade inverted TIRF/epi-fluorescence microscope. The images were recorded on an electron multiplying CCD camera (Andor iXon+ DU-897E). For each sample, over 250 fields of view were acquired. Fluorescent microscopy images were analyzed using a customized MATLAB program based on the SCHNITZCELL segmentation program (a generous gift from M. Elowitz's lab). Images were median filtered to reduce spurious pixel noise. Next, a threshold mask was created for every frame by hand setting a threshold value for each sample (200 for MLG910-Δ*rpoS* strain, 100 for autofluorescence measurement and 500 for the rest of the cells). These masks were used to segment out individual cells from the original images and the total fluorescence of each segmented cell could be recorded. A last stage of manual selection was made on the segmented cells, where the selection criteria were based on morphology. Typically, we retained 800 to 3000 cells for each condition. Histograms were created from the selected cells for each sample.

### Single Molecule Fluorescence Calibration

We calibrated the number of fluorescent counts associated with a single sfGFP protein by measuring the average size of single-step photobleaching events [60]. A reference sample of purified MscL-sfGFP protein (∼10 pM) was loaded into a viewing chamber as described above. In order to maximize signal-to-noise, the fluorescent samples were excited with laser-based TIRF illumination. Movies of the photobleaching molecules were recorded at 4 frames per second. Individual molecules were segmented with a modified version of the MATLAB based PolyParticle-Tracker program [61]. Typical single-molecule time traces are shown in Figures S10A through S10C. The traces were manually selected and fit to step functions. A histogram of counts was constructed and a mean signal was calculated (Figure S10D). The mean value was multiplied by a TIRF/epi-fluorescence calibration factor (described below). The number of MscL subunits per cell was obtained by dividing the total fluorescence signal from a given cell by mean fluorescence counts from a single sfGFP molecule. To determine the number of channels, we assumed there were five subunits per channel.

### TIRF/Epi-fluorescence Calibration

In order to use a signal from single molecules recorded in TIRF to calibrate the signal from single cell recorded in epi-fluorescence, we collected additional set of data used to calculate what we call TIRF/epi-fluorescence calibration factor. A solution of 40 nm yellow-green fluorescent microspheres (Invitrogen F8771) was prepared for imaging as described earlier. The images of 50 fields of view were saved in both TIRF and epi-fluorescence. The PolyParticle-Tracker program was used to find a TIRF/epi-fluorescence ratio for single beads.

## Supporting Information

Figure S1 **Osmotic shock assay results for various strains.** (A) Images of plates comparing cell survival rates after a 0.5 M NaCl osmotic downshift for MG1655, MJF612 and MJF612 expressing MscL-sfGFP (MJF612+*mscL*-*sfGFP*). Rows represent serial dilutions, where the dilution factors are shown to the left of the rows of the first panel. The dilutions factors are relative to the previous row. Columns represent repetitions of the same sample. (B) Mean survival rate determined from the last plate row for the various strains for a given osmotic shock. The results are normalized to colony forming units from the MG1655 strain. The errors bars are the standard deviation of 5 trials.
(TIF)

Figure S2 **Confocal images demonstrating FRAP for single cell.** (A) Before photo-bleaching (0 s). (B) Photo-bleaching (0.7 s) (C) Recovery of the signal (8.4 s). The slow recovery of fluorescence is consistent with diffusion rates typical of fluorescent proteins mobile in the cell membrane, as opposed to the sub-second recovery times which are characteristic of free proteins expressed in the cytoplasm.
(TIF)

Figure S3 **Detection of MscL and MscL-sfGFP with the same antibody.** Arrows indicate the protein of interest. Other bands are the result of non-specific binding. Lysate from MJF612 strain (612, lane 6) was used as a negative control. The cells used for lysate preparations were cultured to exponential (exp) and stationary phase (stat) in LB-Miller (LB), M9+glucose (U), and M9+ glycerol (Y). For the reference lanes (1–5), the numbers near the reference bands are the number of picomoles loaded. For the lysate lanes (7–18), the numbers near the lysate bands are the number of picomoles determined by extrapolation from the reference band intensities. (A) Blot performed with MscL antibody. Lanes 1–5 are reference loads of purified MscL protein. Lanes 7–9 and 13–15 are MscL-sfGFP levels in MLG910 (MLG). Lanes 10–12 and 16–18 are MscL levels in MG1655 (WT). (B) Blot performed with GFP antibody. Lanes 1–5 are reference loads of purified MscL-sfGFP proteins. Lanes 7–9 and 13–15 are MscL-sfGFP levels in MLG910 (MLG). Lanes 10–12 and 16–18 are samples from MG1655 expressing WT MscL and are additional negative controls for the GFP antibody. Comparison of lanes 7–9 and 13–15 from both blots demonstrate the equivalency of the two antibodies.
(TIF)

Figure S4 **Mean number of MscL-sfGFP channels as a function of OD in MLG910-Δ** ***rpoS*** **strain.** For M9+glycerol+500 mM NaCl, we observed a doubling of the mean number of channels. The errors bars reflect the uncertainties in the absolute calibration.
(TIF)

Figure S5 **MG1655 (WT) and MLG910-ΔrpoS strains subjected to a 0.5 M NaCl osmotic shock.** (A) Images of plating results after hypo-osmotic challenge. Rows represent serial dilutions, where the dilution factors are shown to the left of the rows of the first panel. The dilutions factors are relative to the previous row. Columns represent repetitions of the same sample. (B) Mean probability of survival determined from the number of colony forming units after an overnight incubation. The results are normalized to colony forming units from the control plate (no shock). Errors bars are the standard deviation of five trials.
(TIF)

Figure S6 **Fusion protein design and sequence.** The single letter amino acid sequence for MscL, the linker, and sfGFP are colored in brown, pink, and (fluorescent) green, respectively.
(TIF)

Figure S7 **Chromosomal integration strategy.** (A) 23.01 Forward Primer and 23.01R Reverse Primer used for integration. (B) Auxiliary plasmid pZS4*-em7-galK. 4* denotes spectinomycin resistance gene, em7 is a synthetic prokaryotic promoter, and *galK* is the galactokinase coding gene. (C) Native MscL coding region in *E. coli*. (D) 4*-em7-galK cassette inserted into MscL coding region. (E) MscL-sfGFP fusion inserted into MscL coding region. The gene is under control of the native MscL promoter.
(TIF)

Figure S8 **Protein loss during lysate preparation.** Western blots were performed with MscL antibodies. The aliquots from different stages of mock lysate preparation (purified MscL protein of known concentration added to a MJF612 culture) were run in duplicate alongside with the reference protein loads (References). The number above reference each band is the number of picomoles loaded. The aliquots (right to left) were taken after adding lysis buffer to pelleted MJF612 cells (AL), after using a homogenizer (AH), and after passing the sample through a microfluidizer (AF). The numbers above the lysate bands show the number of picomoles measured based on extrapolation from the reference band intensities. Ideally, the mock lysate bands (lanes 4 through 6) should have the same intensity as the 0.675 picomole reference band (lane 3). The other bands are results of non-specific binding.
(TIF)

Figure S9 **Absolute calibration of quantitative Western blots.** (A) Representative bands for MG1655 (WT) with a summary table of measured and calculated numbers. (B) Linear fit to reference band intensities used to determine the number of channels for each lysate. Only the linear range of detection is used for this fit (lanes 2 through 5). All of the lysate band intensities (Figure S9A row 1, lanes 7 through 12) occur within this range. The last data point, demonstrating saturation effects from lane 1, has been excluded from the fit.
(TIF)

Figure S10 **Single molecule calibration: typical single molecule traces and histogram of counts.** (A) A trace with one photobleaching step. (B) A region of the trace with two photobleaching steps. The data points not used for fitting are marked in blue. (C) A typical single molecule trace that was rejected. (D) Histogram of counts.
(TIF)

Supporting Information S1 **Supporting information related to** **Materials and Methods** **.**
(DOC)

Table S1 **Osmolalities of the media used to culture the cells.**
(DOC)

Table S2 **A summary of results for channels counts and gamma distribution fitting.**
(DOC)

Table S3 **Cell density calibration of OD_600_ measurements for various growth conditions.**
(DOC)
