# Supplementary figures and images for: Single-Cell Census of Mechanosensitive Channels in Living Bacteria
Source: PLoS One. 2012 Mar 13;7(3):e33077. doi: 10.1371/journal.pone.0033077 (PMC3302805; doi:10.1371/journal.pone.0033077)

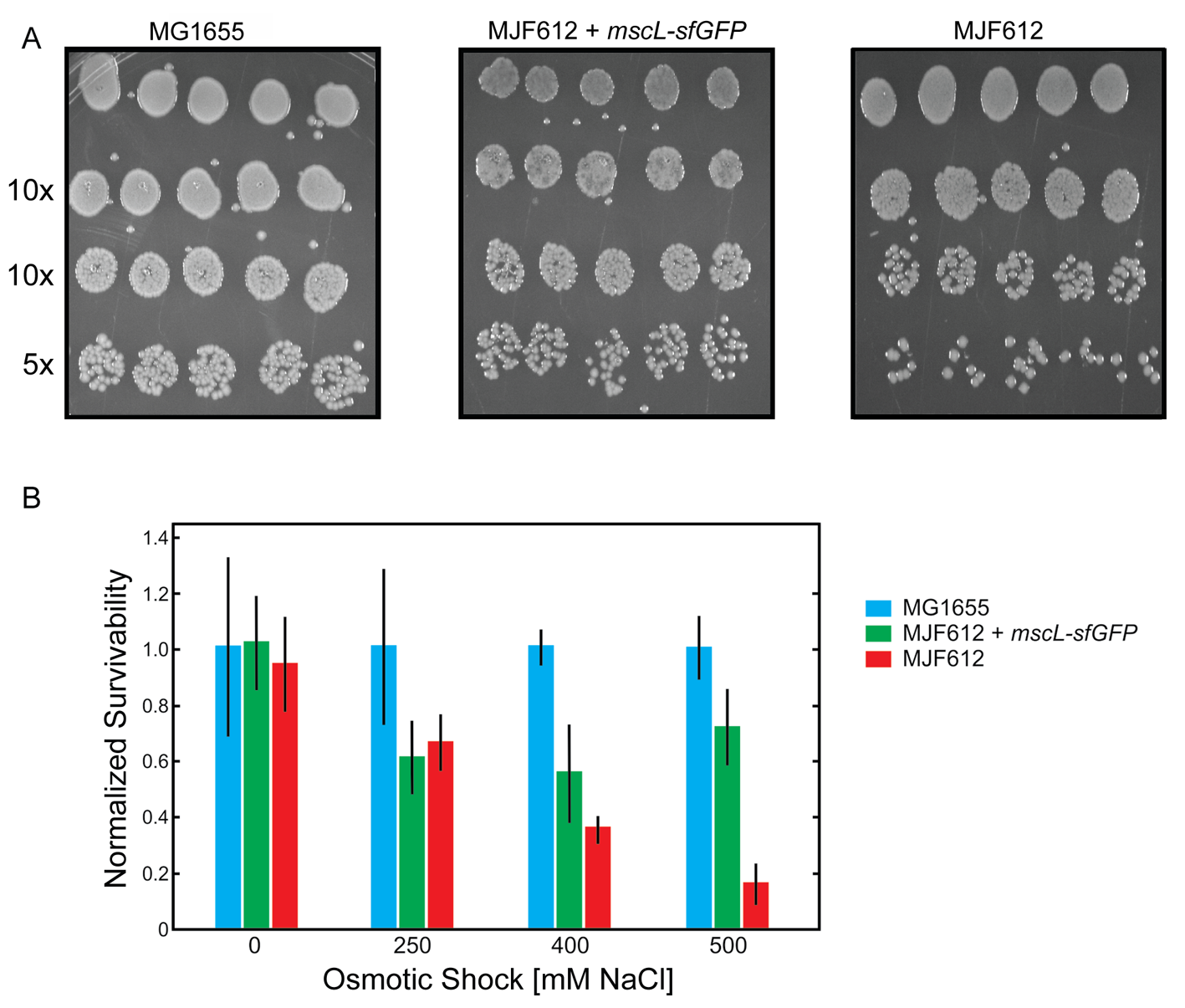

Supplement: Figure S1 — Osmotic shock assay results for various strains. (A) Images of plates comparing cell survival rates after a 0.5 M NaCl osmotic downshift for MG1655, MJF612 and MJF612 expressing MscL-sfGFP (MJF612+mscL-sfGFP). Rows represent serial dilutions, where the dilution factors are shown to the left of the rows of the first panel. The dilutions factors are relative to the previous row. Columns represent repetitions of the same sample. (B) Mean survival rate determined from the last plate row for the various strains for a given osmotic shock. The results are normalized to colony forming units from the MG1655 strain. The errors bars are the standard deviation of 5 trials. (TIF) [file pone.0033077.s001.tif]

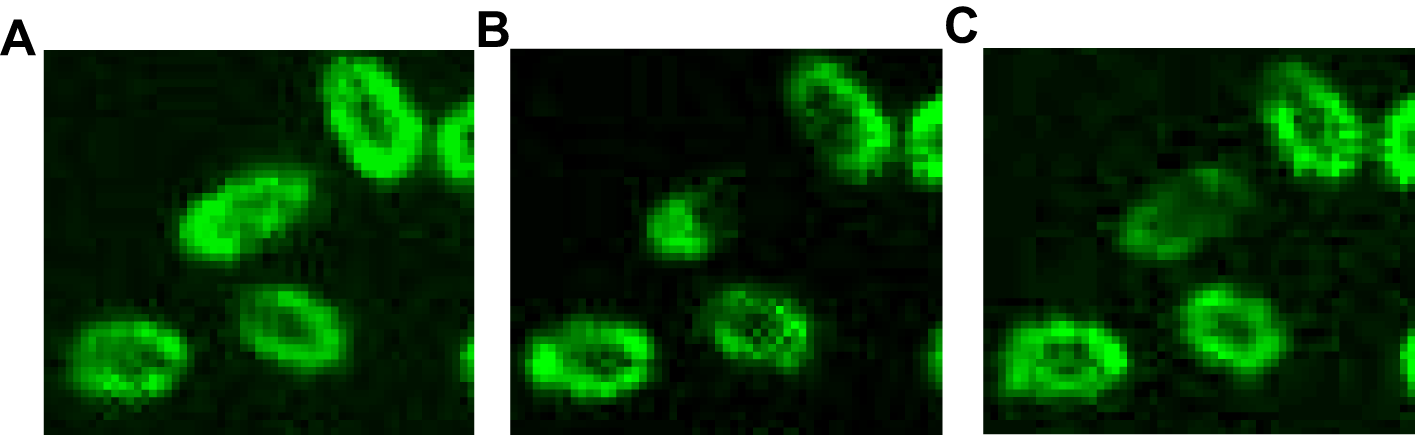

Supplement: Figure S2 — Confocal images demonstrating FRAP for single cell. (A) Before photo-bleaching (0 s). (B) Photo-bleaching (0.7 s) (C) Recovery of the signal (8.4 s). The slow recovery of fluorescence is consistent with diffusion rates typical of fluorescent proteins mobile in the cell membrane, as opposed to the sub-second recovery times which are characteristic of free proteins expressed in the cytoplasm. (TIF) [file pone.0033077.s002.tif]

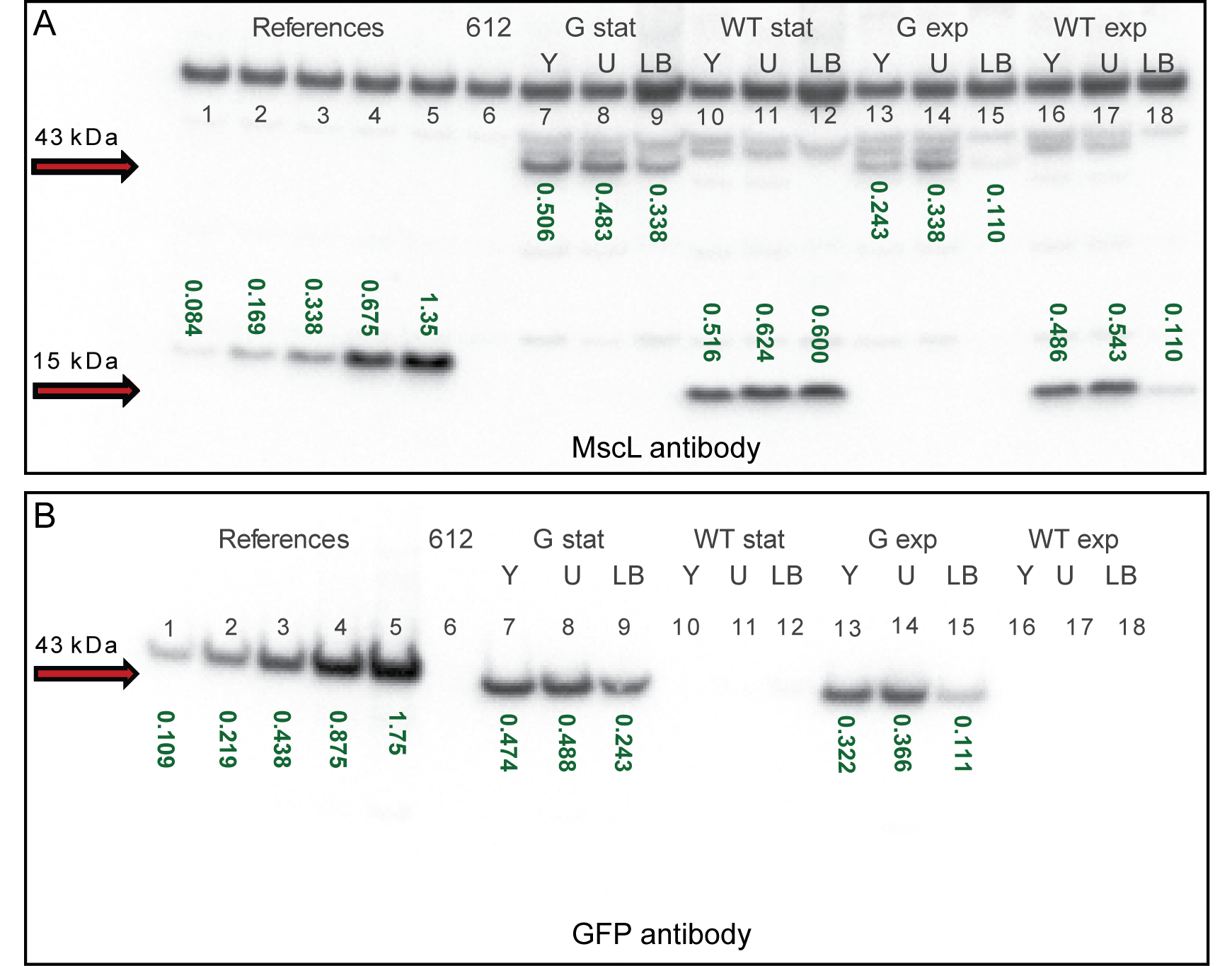

Supplement: Figure S3 — Detection of MscL and MscL-sfGFP with the same antibody. Arrows indicate the protein of interest. Other bands are the result of non-specific binding. Lysate from MJF612 strain (612, lane 6) was used as a negative control. The cells used for lysate preparations were cultured to exponential (exp) and stationary phase (stat) in LB-Miller (LB), M9+glucose (U), and M9+ glycerol (Y). For the reference lanes (1–5), the numbers near the reference bands are the number of picomoles loaded. For the lysate lanes (7–18), the numbers near the lysate bands are the number of picomoles determined by extrapolation from the reference band intensities. (A) Blot performed with MscL antibody. Lanes 1–5 are reference loads of purified MscL protein. Lanes 7–9 and 13–15 are MscL-sfGFP levels in MLG910 (MLG). Lanes 10–12 and 16–18 are MscL levels in MG1655 (WT). (B) Blot performed with GFP antibody. Lanes 1–5 are reference loads of purified MscL-sfGFP proteins. Lanes 7–9 and 13–15 are MscL-sfGFP levels in MLG910 (MLG). Lanes 10–12 and 16–18 are samples from MG1655 expressing WT MscL and are additional negative controls for the GFP antibody. Comparison of lanes 7–9 and 13–15 from both blots demonstrate the equivalency of the two antibodies. (TIF) [file pone.0033077.s003.tif]

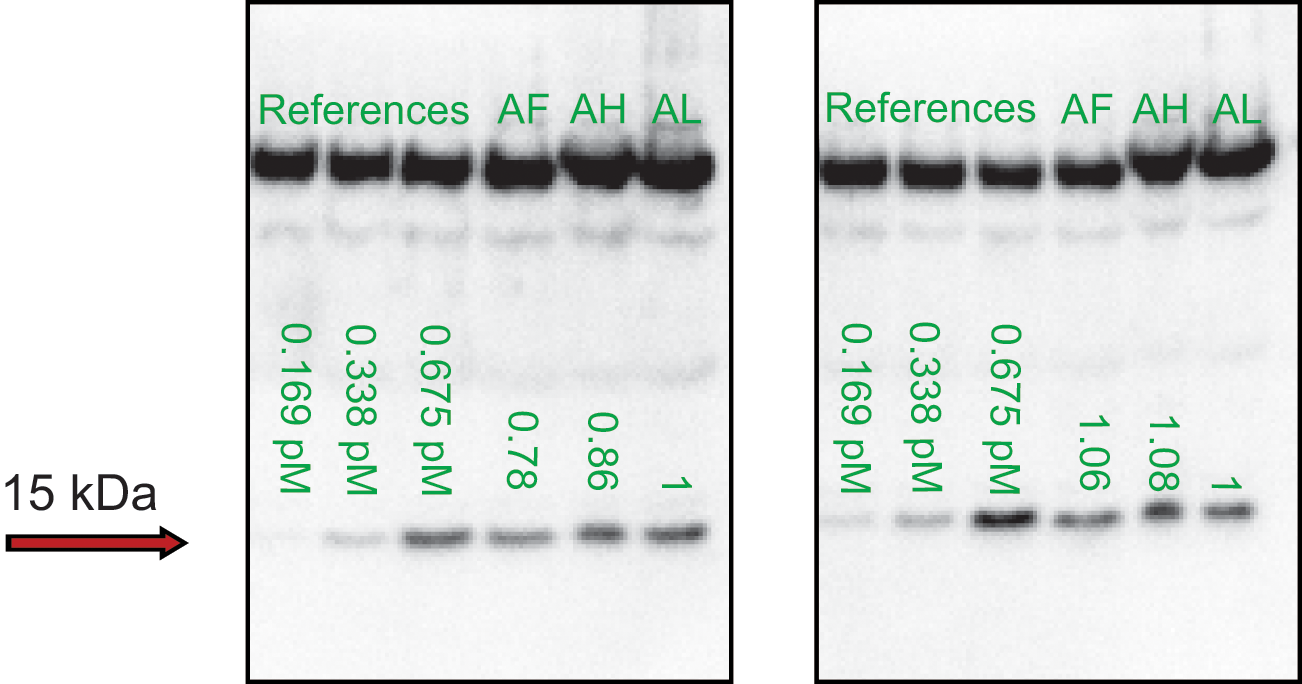

Supplement: Figure S4 — Mean number of MscL-sfGFP channels as a function of OD in MLG910-Δ rpoS strain. For M9+glycerol+500 mM NaCl, we observed a doubling of the mean number of channels. The errors bars reflect the uncertainties in the absolute calibration. (TIF) [file pone.0033077.s004.tif]

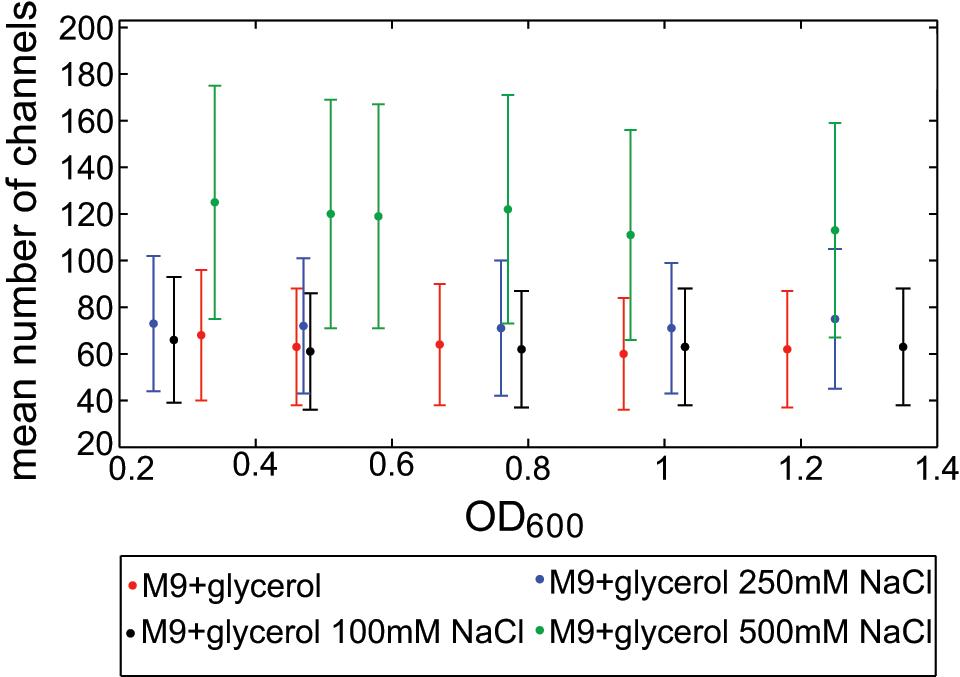

Supplement: Figure S5 — MG1655 (WT) and MLG910-ΔrpoS strains subjected to a 0.5 M NaCl osmotic shock. (A) Images of plating results after hypo-osmotic challenge. Rows represent serial dilutions, where the dilution factors are shown to the left of the rows of the first panel. The dilutions factors are relative to the previous row. Columns represent repetitions of the same sample. (B) Mean probability of survival determined from the number of colony forming units after an overnight incubation. The results are normalized to colony forming units from the control plate (no shock). Errors bars are the standard deviation of five trials. (TIF) [file pone.0033077.s005.tif]

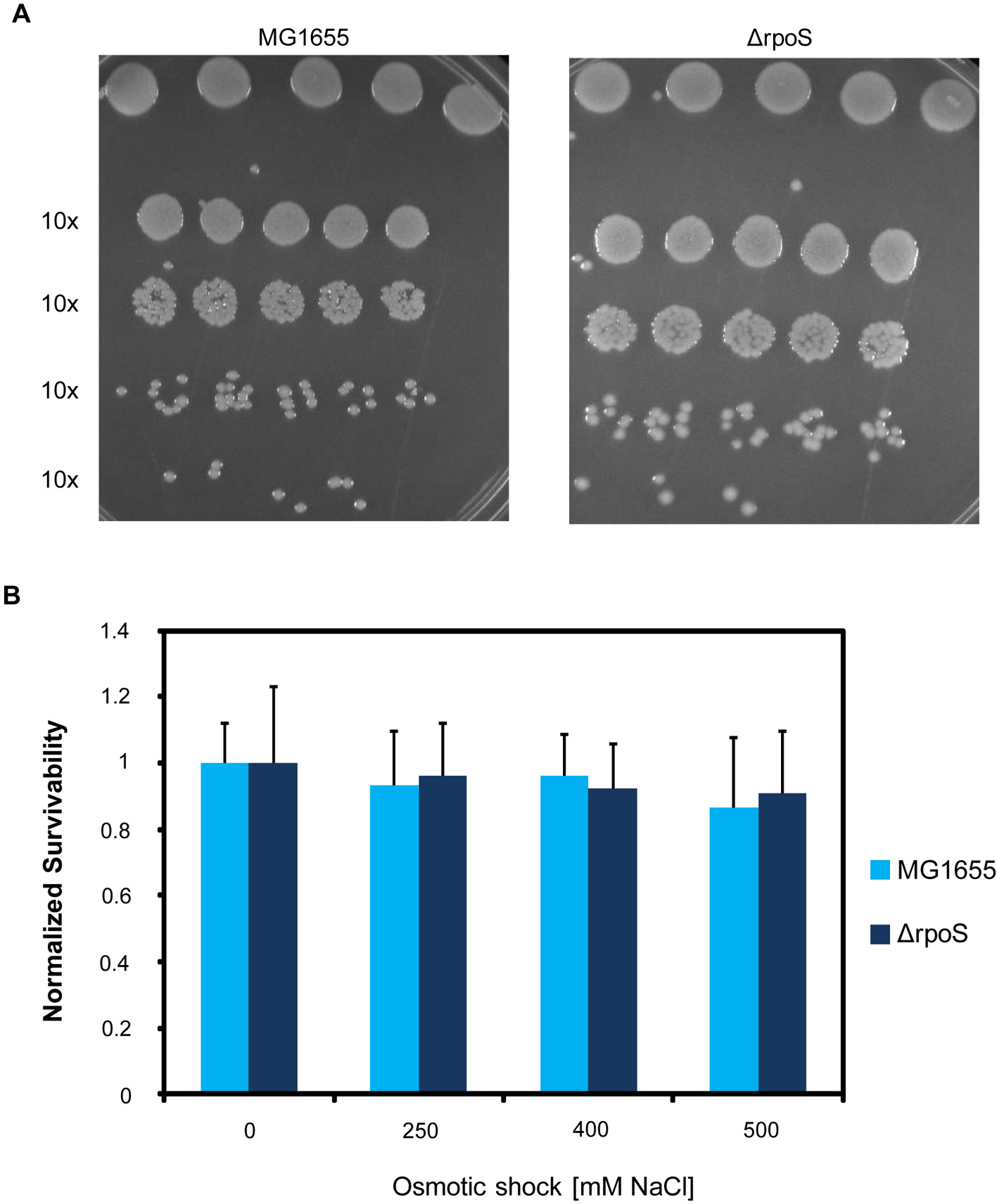

Supplement: Figure S6 — Fusion protein design and sequence. The single letter amino acid sequence for MscL, the linker, and sfGFP are colored in brown, pink, and (fluorescent) green, respectively. (TIF) [file pone.0033077.s006.tif]

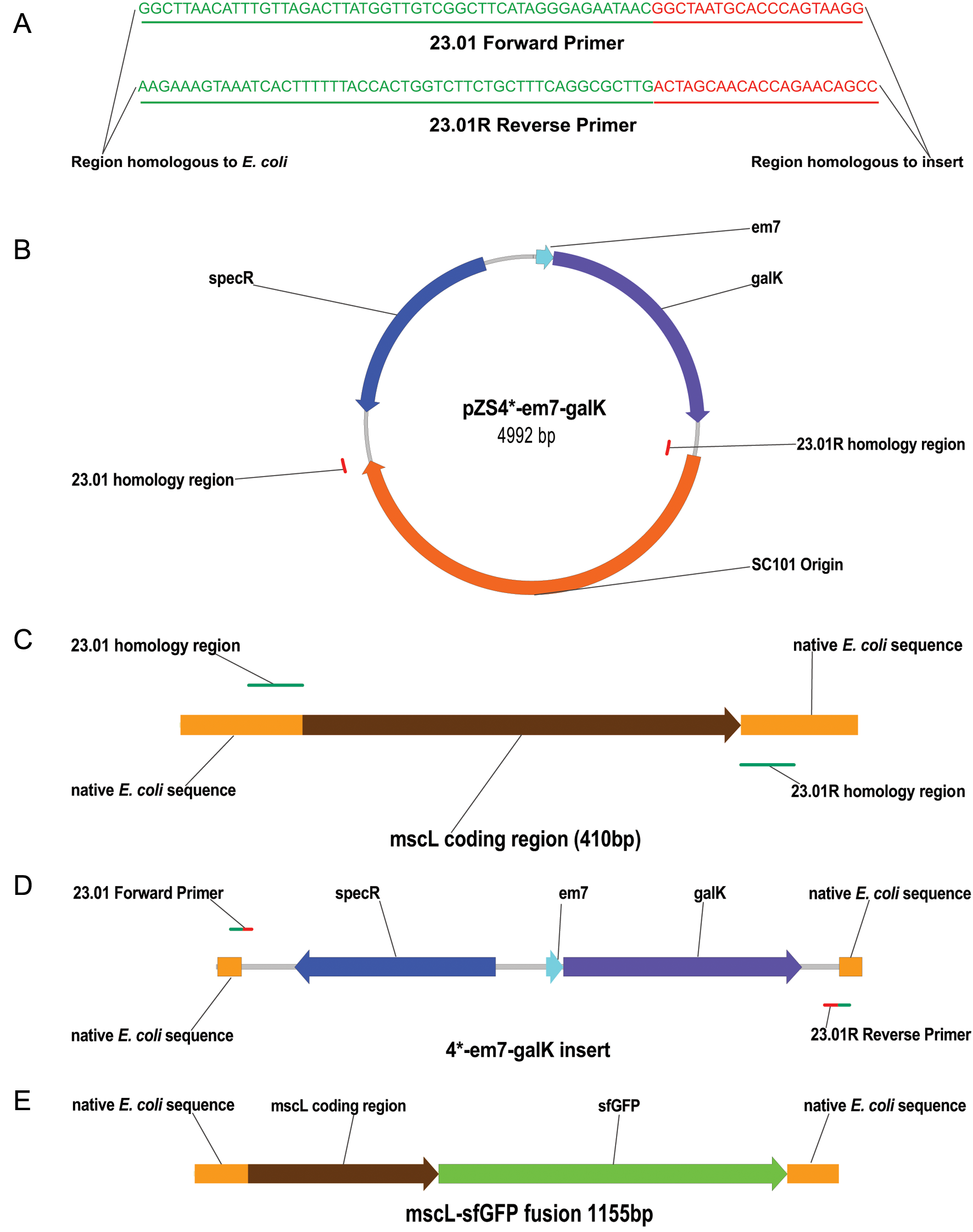

Supplement: Figure S7 — Chromosomal integration strategy. (A) 23.01 Forward Primer and 23.01R Reverse Primer used for integration. (B) Auxiliary plasmid pZS4*-em7-galK. 4* denotes spectinomycin resistance gene, em7 is a synthetic prokaryotic promoter, and galK is the galactokinase coding gene. (C) Native MscL coding region in E. coli. (D) 4*-em7-galK cassette inserted into MscL coding region. (E) MscL-sfGFP fusion inserted into MscL coding region. The gene is under control of the native MscL promoter. (TIF) [file pone.0033077.s007.tif]

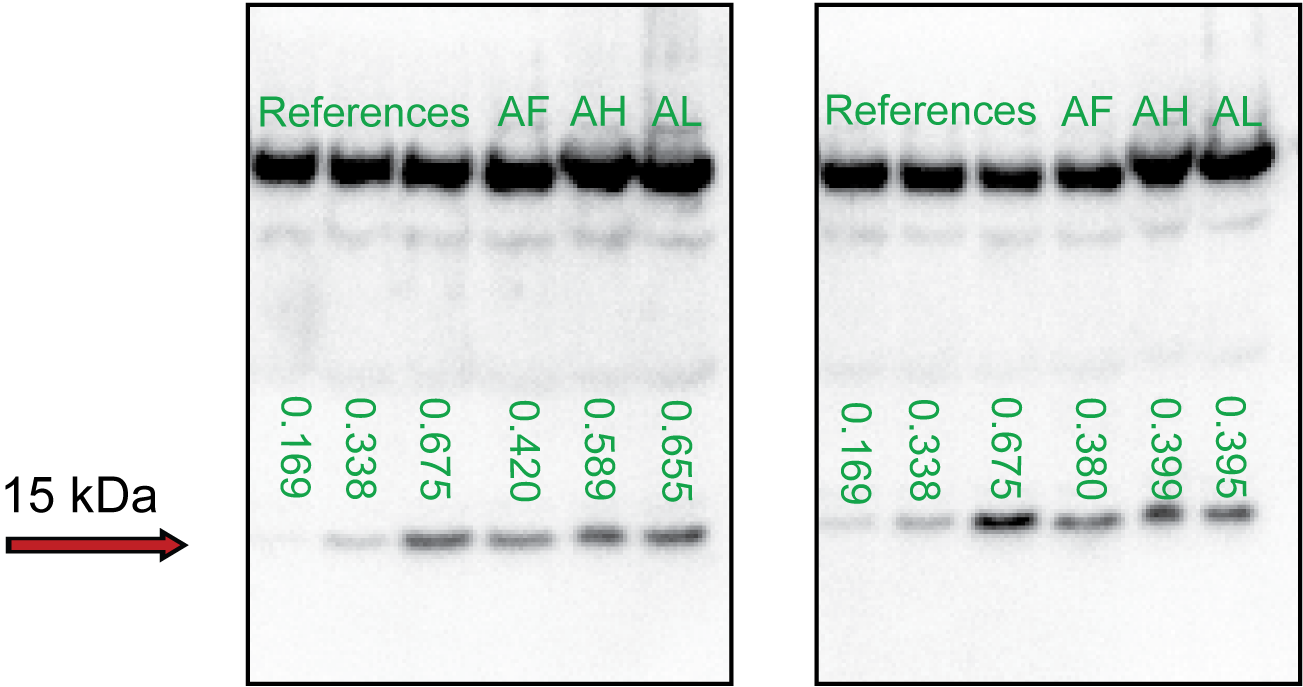

Supplement: Figure S8 — Protein loss during lysate preparation. Western blots were performed with MscL antibodies. The aliquots from different stages of mock lysate preparation (purified MscL protein of known concentration added to a MJF612 culture) were run in duplicate alongside with the reference protein loads (References). The number above reference each band is the number of picomoles loaded. The aliquots (right to left) were taken after adding lysis buffer to pelleted MJF612 cells (AL), after using a homogenizer (AH), and after passing the sample through a microfluidizer (AF). The numbers above the lysate bands show the number of picomoles measured based on extrapolation from the reference band intensities. Ideally, the mock lysate bands (lanes 4 through 6) should have the same intensity as the 0.675 picomole reference band (lane 3). The other bands are results of non-specific binding. (TIF) [file pone.0033077.s008.tif]

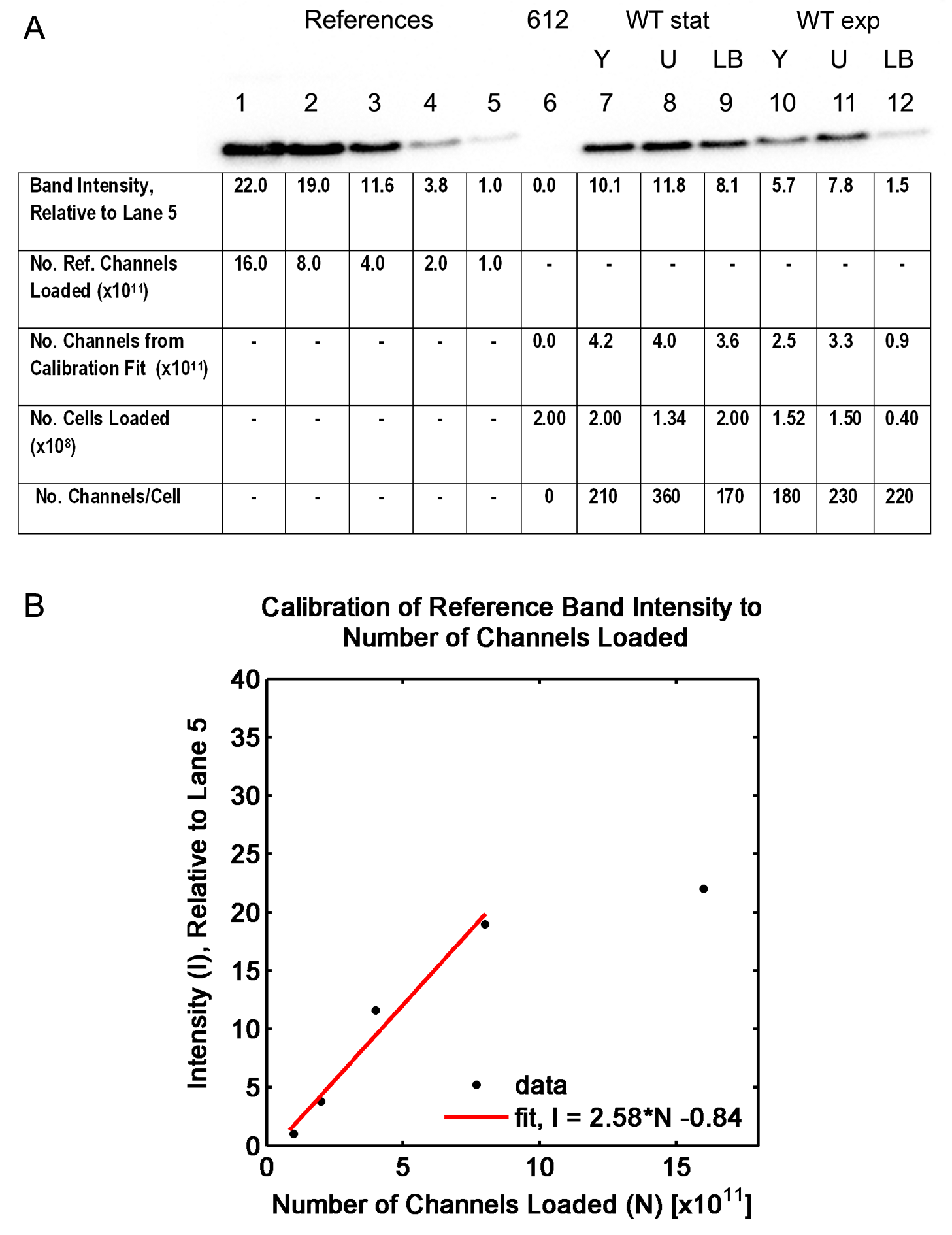

Supplement: Figure S9 — Absolute calibration of quantitative Western blots. (A) Representative bands for MG1655 (WT) with a summary table of measured and calculated numbers. (B) Linear fit to reference band intensities used to determine the number of channels for each lysate. Only the linear range of detection is used for this fit (lanes 2 through 5). All of the lysate band intensities (Figure S9A row 1, lanes 7 through 12) occur within this range. The last data point, demonstrating saturation effects from lane 1, has been excluded from the fit. (TIF) [file pone.0033077.s009.tif]

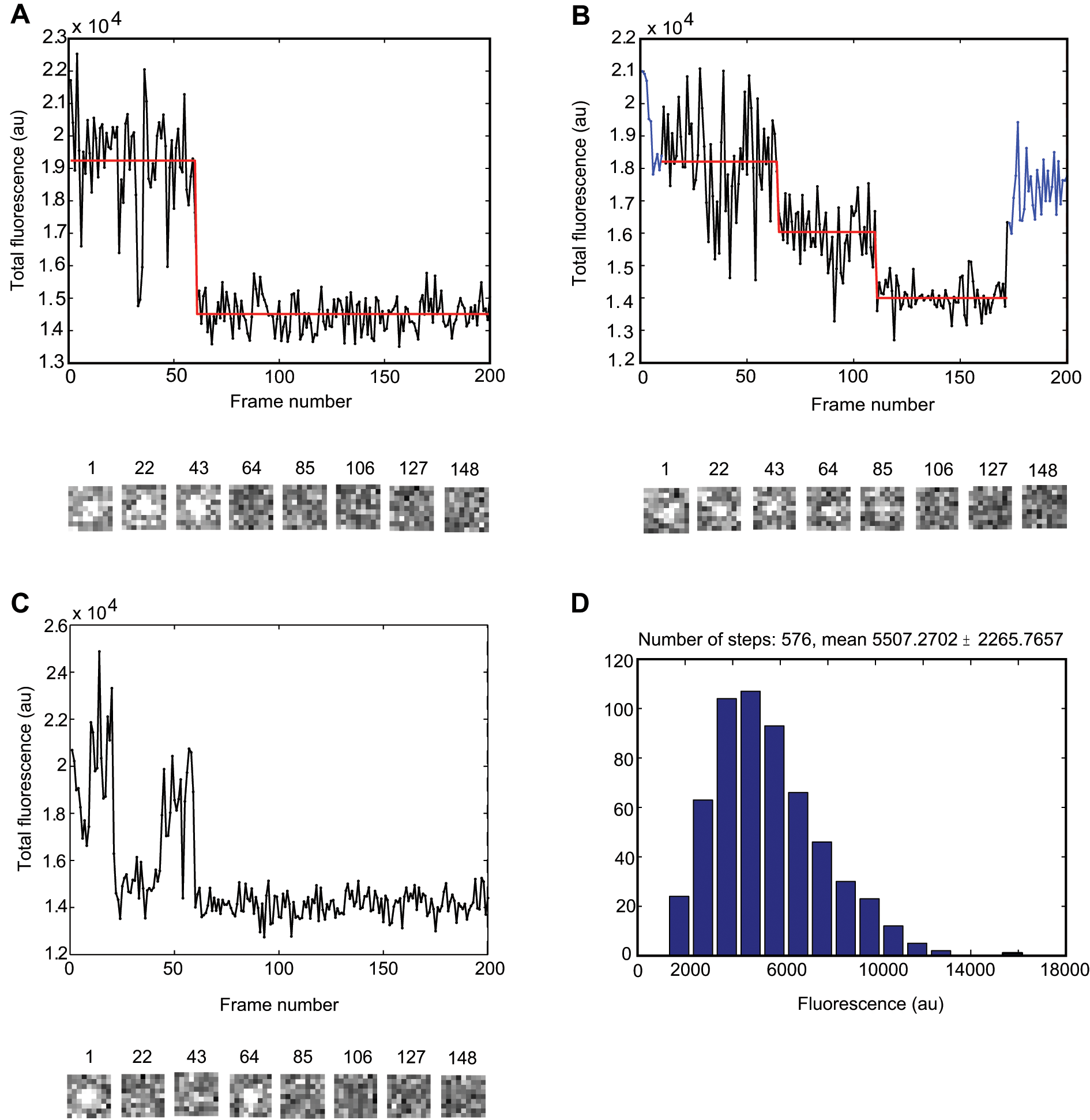

Supplement: Figure S10 — Single molecule calibration: typical single molecule traces and histogram of counts. (A) A trace with one photobleaching step. (B) A region of the trace with two photobleaching steps. The data points not used for fitting are marked in blue. (C) A typical single molecule trace that was rejected. (D) Histogram of counts. (TIF) [file pone.0033077.s010.tif]
