# Supplementary material for: Single-Cell Census of Mechanosensitive Channels in Living Bacteria
Source: PLoS One. 2012 Mar 13;7(3):e33077. doi: 10.1371/journal.pone.0033077 (PMC3302805; doi:10.1371/journal.pone.0033077)
Supplement: Supporting Information S1 — Supporting information related to Materials and Methods . (DOC) [file pone.0033077.s011.doc]

**Supporting Information for Materials and Methods**

**Plaque/Survivability Assays**

In order to test the functionality of our construct, we performed a survivability assay. 5 mL LB-Miller cultures of desired strains were grown aerobically at 37°C to an OD_600_ range of 0.3 - 0.4 in the presence of antibiotics and, afterwards, were then diluted (1:1000) in 0.5 M NaCl LB-Miller and grown to OD_600_ 0.25 – 0.35. Cells were diluted serially 10-fold in shock media (0.25 M NaCl, 0.1 M NaCl, and 0 M NaCl LB-Miller) and after each dilution 5 μL of the diluted culture were plated in 5 repetitions on LB-Miller plates of osmolality corresponding to the osmolality of the shock media. As a control, cells were serially diluted in media of the same osmolarity and plated on 0.5 M NaCl LB-Miller plates. Plates were incubated at 37°C overnight and the next morning colonies were counted (Figure S1A). The survival rates of these strains are presented in Figure S1B.

**Fusion Protein Design and Sequence**

Given the relative size of sfGFP to MscL and its proximity to the MscL C-terminal bundle, we incorporated an amino acid linker in our fusion protein to reduce steric hindrance effects that could affect proper assembly and/or function of complete channels [S1]. We inserted a flexible linker of 11 amino acids (SASGENAARGH) between the MscL C-terminus and the sfGFP N-terminus (Figure S6).

**Chromosomal Integration Strategy**

The native MscL sequence was replaced by MscL-sfGFP sequence without the addition of an antibiotic resistance marker. To do this, we used a negative selection scheme based on the eventual absence of the *galK* gene [S2]. Preparation of the intermediate strain for the fusion integration requires deletion of the native *galK* gene and re-inserting an auxiliary *galK* gene into the MscL region. Successful integration of the fusion in to the MscL region will remove the auxiliary *galK* gene. First, the native *galK* gene in MG1655 was deleted by P1 transduction [S3] from an auxiliary strain (HG105-KD4) which carried the deletion (Δ*galK::* kanamycin). Next, we used adapter primers 23.01 and 23.01R (Figure S7A) to generate an auxiliary fragment from the pZS4*em7-galK plasmid (Figure S7B) by PCR. Each primer had a region that was homologous to the plasmid and a 50bp region that was homologous to the native *E. coli* sequence in the MscL region (Figure S7C). The coding region of MscL was replaced with this fragment by homologous recombination, leading to the insertion of the *galK* and *specR* genes (Figure S7D). Successful integration of this fragment was selected for by spectinomycin resistance in liquid culture. The strain carrying the auxiliary fragment in the MscL region is the competent strain for the eventual final integration. After a second round of homologous recombination, the integrated auxiliary fragment was replaced with the fusion construct (Figure S7E). Successful integration of the fusion construct was negatively selected for by the absence of the *galK* gene by growth on minimal media plates supplemented with 2-deoxy galactose. Single colonies generated from the 2-deoxy galactose plates were screened by streaking a given colony on a LB-Miller plate carrying kanamycin and a LB-Miller plate carrying spectinomycin (negative control).

**Protein Loss During Lysate Preparation**

One of the issues that we wanted to examine was the extent to which protein was lost during the process of preparing our Western blot samples. To that end, we performed a mock lysis procedure, where we added purified MscL or MscL-sfGFP protein of known concentration to a resuspension of MJF612 cells before passing the cells through the described lysis procedure. The concentration of protein was set to 33.8nM after the addition of lysis buffer. 20 μL of this dilution (a load of 0.675 picomole) was kept as a no-loss control sample. The rest of the dilution was run through the remaining steps of the lysate preparation protocol. 20 μL aliquots were saved for further analysis after using a homogenizer and after passing the sample through the fluidizer. All aliquots were run on a gel alongside three reference dilutions (Figure S8). The loss of protein was estimated on the basis of comparison of the chemiluminescence intensity of bands representing samples at different stages of lysate preparation with each other and the purified protein reference.

**Calibrating the Cell Density for OD_600_ Measurements**

In order to determine the number of MscL channels per cell for our Western blots, we determined the conversion factor between an OD_600_ measurement and the absolute cell density for a given condition. A 1 mL aliquot of a cell culture of known OD_600_ was diluted 10 to 40-fold in media of similar osmolarity containing FM 4-64 dye (Invitrogen). 10 μL of this cell suspension was loaded into a disposable hemocytometer (C-Chip DHC-S01-2, iNCYTO). Five different regions of the chip were imaged using a 10x objective using both phase and fluorescence microscopy. Cells were manually counted, averaged for 5 fields of view and converted into a number of cells per mL of undiluted cell suspension. The cell densities for cells grown to a specific OD_600_ in different media are listed in Table S3.

References:

S1. Gandhi CS, Walton TA, Rees DC (2011) OCAM: a new tool for studying the oligomeric diversity of MscL channels. Protein Sci 20:313-326.

S2. Warming S, Costantino N, Court D, Jenkins NA, Copeland NG (2005) Simple and highly efficient BAC recombineering using galK selection. Nuc Acids Research 33: e36.

S3. Miller JH (1992) Short Course in Bacterial Genetics: A Laboratory Manual and Handbook for Escherichia coli and Related Bacteria. Cold Spring Harbor Laboratory Press. 357p.
