# Supplementary material for: Single-Cell Census of Mechanosensitive Channels in Living Bacteria
Source: PLoS One. 2012 Mar 13;7(3):e33077. doi: 10.1371/journal.pone.0033077 (PMC3302805; doi:10.1371/journal.pone.0033077)
Supplement: Table S1 — Osmolalities of the media used to culture the cells. (DOC) [file pone.0033077.s012.doc]

**Table S1. Osmolalities of the media used to culture the cells.**

| **Supplemented Salt** | **LB-Miller** | **M9 + glucose** | **M9 + glycerol** |
| --- | --- | --- | --- |
| **0 M NaCl** | 392 | 234 | 277 |
| **0.1 M NaCl** | --- | 342 | 393 |
| **0.25 M NaCl** | --- | 529 | 576 |
| **0.5 M NaCl** | --- | 886 | 934 |

All entries are measured in mOsm/kg. The osmolalities of our various media were measured with a Vapro vapor pressure osmometer model 5520 (Wescor Inc). The manufacturer’s specified absolute error is ±3mOsm/kg.
