# Supplementary material for: Single-Cell Census of Mechanosensitive Channels in Living Bacteria
Source: PLoS One. 2012 Mar 13;7(3):e33077. doi: 10.1371/journal.pone.0033077 (PMC3302805; doi:10.1371/journal.pone.0033077)
Supplement: Table S2 — A summary of results for channels counts and gamma distribution fitting. (DOC) [file pone.0033077.s013.doc]

**Table S2**: **A summary of results for channels counts and gamma distribution fitting.**

| **MLG910** | | | | | | |
| --- | --- | --- | --- | --- | --- | --- |
| *media* | *OD_600_* | *# of cells* | *Mean count* | *Fano factor* | *a* | *b* |
| LB-Miller | 0.33 | 2503 | 340 | 17.6408611 | 6.7152 | 50.6649 |
| LB-Miller | 0.49 | 2556 | 293 | 68.98253 | 6.1215 | 47.8332 |
| LB-Miller | 1.08 | 788 | 330 | 43.49771 | 9.3501 | 35.3103 |
| LB-Miller | 1.27 | 1555 | 320 | 42.77948 | 8.8456 | 36.1941 |
| LB-Miller | 1.74 | 6165 | 472 | 92.64373 | 6.2285 | 75.8225 |
| M9+glucose | 0.3 | 3084 | 466 | 78.78777 | 7.1114 | 65.5902 |
| M9+glucose | 0.51 | 1221 | 466 | 118.3628 | 5.0172 | 92.9789 |
| M9+glucose | 0.67 | 1756 | 552 | 139.3568 | 4.9949 | 110.4617 |
| M9+glucose | 0.95 | 2559 | 560 | 137.6172 | 4.9512 | 113.0242 |
| M9+glucose | 1.23 | 1520 | 746 | 126.0476 | 6.4454 | 115.7735 |
| M9+glucose+0.1M NaCl | 0.29 | 4280 | 780 | 191.23870 | 4.8270 | 161.5811 |
| M9+glucose+0.1M NaCl | 0.52 | 3663 | 802 | 158.5049 | 6.4774 | 123.8357 |
| M9+glucose+0.1M NaCl | 0.7 | 2019 | 776 | 130.4718 | 7.2173 | 107.5843 |
| M9+glucose+0.1M NaCl | 1 | 3799 | 786 | 141.1716 | 7.1582 | 109.7980 |
| M9+glucose+0.1M NaCl | 1.43 | 4397 | 1234 | 237.9579 | 6.5788 | 187.5706 |
| M9+glucose+0.25M NaCl | 0.25 | 3429 | 358 | 69.73346 | 6.7773 | 52.8596 |
| M9+glucose+0.25M NaCl | 0.46 | 3976 | 951 | 192.7966 | 6.5631 | 144.9569 |
| M9+glucose+0.25M NaCl | 0.71 | 5971 | 956 | 196.7224 | 6.4514 | 148.2273 |
| M9+glucose+0.25M NaCl | 1.01 | 3569 | 985 | 212.4989 | 6.3969 | 153.9933 |
| M9+glucose+0.25M NaCl | 1.21 | 2684 | 1314 | 240.0539 | 6.9047 | 190.3492 |
| M9+glucose+0.5M NaCl | 0.26 | 1681 | 1419 | 227.7141 | 7.9413 | 178.6636 |
| M9+glucose+0.5M NaCl | 0.35 | 2261 | 1585 | 331.3018 | 6.1975 | 255.8249 |
| M9+glucose+0.5M NaCl | 0.45 | 2540 | 1324 | 215.3837 | 8.0087 | 165.2828 |
| M9+glucose+0.5M NaCl | 0.71 | 3921 | 1422 | 241.854 | 7.4901 | 189.8175 |
| M9+glucose+0.5M NaCl | 0.91 | 1546 | 1394 | 276.4679 | 6.7176 | 207.5572 |
| M9+glucose+0.5M NaCl | 1.2 | 1503 | 1353 | 235.0474 | 7.2670 | 186.2539 |
| M9+glycerol | 0.31 | 2098 | 709 | 112.7239 | 8.2344 | 86.1558 |
| M9+glycerol | 0.41 | 4635 | 686 | 123.8411 | 6.6806 | 102.7219 |
| M9+glycerol | 0.62 | 3248 | 681 | 128.9213 | 6.3772 | 106.8403 |
| M9+glycerol | 0.85 | 4385 | 747 | 150.5265 | 5.9535 | 125.5369 |
| M9+glycerol | 1 | 3227 | 680 | 141.0444 | 6.2205 | 109.3368 |
| M9+glycerol | 1.15 | 4960 | 721 | 145.3868 | 6.1545 | 117.0988 |
| M9+glycerol | 1.27 | 3914 | 729 | 159.2388 | 6.2790 | 116.1648 |
| M9+glycerol+0.1M NaCl | 0.4 | 3923 | 870 | 184.3223 | 6.2537 | 139.1729 |
| M9+glycerol+0.1M NaCl | 0.76 | 2631 | 827 | 144.6099 | 7.4902 | 110.3775 |
| M9+glycerol+0.1M NaCl | 1.07 | 1949 | 847 | 129.1219 | 8.5798 | 98.7149 |
| M9+glycerol+0.25M NaCl | 0.19 | 3712 | 1013 | 181.4919 | 6.9965 | 144.7469 |
| M9+glycerol+0.25M NaCl | 0.28 | 2426 | 1173 | 228.9686 | 6.6479 | 176.4324 |
| M9+glycerol+0.25M NaCl | 0.33 | 4073 | 1043 | 237.7741 | 5.8768 | 177.4560 |
| M9+glycerol+0.25M NaCl | 0.46 | 2258 | 951 | 192.7966 | 8.1167 | 105.7527 |
| M9+glycerol+0.25M NaCl | 0.56 | 2348 | 1125 | 251.1459 | 5.9777 | 188.1924 |
| M9+glycerol+0.25M NaCl | 0.68 | 1371 | 875 | 149.4666 | 7.7279 | 113.2247 |
| M9+glycerol+0.25M NaCl | 0.71 | 2248 | 866 | 170.2669 | 7.8821 | 109.8916 |
| M9+glycerol+0.25M NaCl | 0.92 | 1497 | 865 | 194.9382 | 6.9831 | 123.8102 |
| M9+glycerol+0.25M NaCl | 1.26 | 2267 | 938 | 168.3297 | 7.5704 | 123.9285 |
| M9+glycerol+0.5M NaCl | 0.19 | 3351 | 1746 | 350.3273 | 6.1965 | 281.7584 |
| M9+glycerol+0.5M NaCl | 0.21 | 3531 | 1511 | 337.0336 | 5.8162 | 259.7950 |
| M9+glycerol+0.5M NaCl | 0.23 | 3230 | 1634 | 287.2615 | 7.3157 | 223.3389 |
| M9+glycerol+0.5M NaCl | 0.46 | 1226 | 1423 | 261.1942 | 6.7470 | 210.8849 |
| M9+glycerol+0.5M NaCl | 0.5 | 2520 | 1508 | 331.159 | 6.4267 | 236.8047 |
| M9+glycerol+0.5M NaCl | 0.71 | 2727 | 1709 | 425.3123 | 5.0346 | 339.4075 |
| M9+glycerol+0.5M NaCl | 0.83 | 1366 | 1348 | 228.5577 | 7.2754 | 185.2823 |
| M9+glycerol+0.5M NaCl | 0.99 | 1689 | 1501 | 216.2896 | 8.5913 | 174.6689 |
| M9+glycerol+0.5M NaCl | 1.23 | 1253 | 1443 | 211.2783 | 8.7255 | 165.4151 |
| **MLG910-Δ*rpoS*** | | | | | | |
| *media* | *OD_600_* | *# of cells* | *Mean count* | *Fano factor* | *a* | *b* |
| LB-Miller | 0.24 | 1429 | 108 | 45.0497 | 6.0750 | 17.8172 |
| LB-Miller | 0.42 | 1496 | 109 | 19.63285 | 6.1966 | 17.6530 |
| LB-Miller | 0.65 | 2256 | 80 | 16.39978 | 6.1865 | 12.8821 |
| LB-Miller | 0.86 | 2677 | 89 | 19.31024 | 6.4813 | 13.7722 |
| LB-Miller | 1.01 | 2555 | 95 | 16.09938 | 7.2978 | 12.9749 |
| LB-Miller | 1.2 | 2783 | 115 | 24.8767 | 6.5511 | 17.5012 |
| M9+glucose | 0.46 | 1444 | 64 | 8.742002 | 8.7053 | 7.3483 |
| M9+glucose | 0.6 | 3043 | 65 | 8.490565 | 8.8846 | 7.3159 |
| M9+glucose | 0.81 | 2179 | 66 | 8.306852 | 9.2411 | 7.1535 |
| M9+glucose | 0.96 | 2560 | 56 | 9.410702 | 7.2931 | 7.6971 |
| M9+glucose | 1.18 | 3785 | 58 | 12.48343 | 5.9048 | 9.8834 |
| M9+glycerol | 0.32 | 2932 | 68 | 10.20687 | 8.3619 | 8.1879 |
| M9+glycerol | 0.46 | 1367 | 63 | 8.814788 | 9.2509 | 6.8037 |
| M9+glycerol | 0.67 | 1100 | 64 | 9.418268 | 9.0937 | 7.0203 |
| M9+glycerol | 0.94 | 1000 | 60 | 7.645006 | 9.7265 | 6.1819 |
| M9+glycerol | 1.18 | 1182 | 62 | 7.23113 | 10.4019 | 6.0069 |
