# Supplementary material for: Single-Cell Census of Mechanosensitive Channels in Living Bacteria
Source: PLoS One. 2012 Mar 13;7(3):e33077. doi: 10.1371/journal.pone.0033077 (PMC3302805; doi:10.1371/journal.pone.0033077)
Supplement: Table S3 — Cell density calibration of OD600 measurements for various growth conditions. (DOC) [file pone.0033077.s014.doc]

**Table S3**. **Cell density calibration of OD600 for various growth conditions.**

| **Strain** | **Growth Phase** | **Media** | **OD_600_** | **Cell Density (x10^8^ cm^-3^)** |
| --- | --- | --- | --- | --- |
| MG1655 | Exponential | LB-Miller | 0.30 | 0.8 |
| MG1655 | Exponential | M9+glucose | 0.30 | 2.2 |
| MG1655 | Exponential | M9+glycerol | 0.30 | 2.3 |
| MLG910 | Exponential | LB-Miller | 0.30 | 0.8 |
| MLG910 | Exponential | M9+glucose | 0.30 | 2.2 |
| MLG910 | Exponential | M9+glycerol | 0.31 | 2.4 |
| MG1655 | Stationary | LB-Miller | 1.78 | 17.8 |
| MG1655 | Stationary | M9+glucose | 1.36 | 9.1 |
| MG1655 | Stationary | M9+glycerol | 1.24 | 12.4 |
| MLG910 | Stationary | LB-Miller | 1.72 | 17.2 |
| MLG910 | Stationary | M9+glucose | 1.34 | 9.0 |
| MLG910 | Stationary | M9+glycerol | 1.17 | 11.7 |
| MLG910-Δ*rpoS* | Exponential | LB-Miller | 0.30 | 0.81 |
| MLG910-Δ*rpoS* | Exponential | M9+glucose | 0.30 | 3.0 |
| MLG910-Δ*rpoS* | Exponential | M9+glycerol | 0.30 | 3.0 |
| MLG910-Δ*rpoS* | Stationary | LB-Miller | 1.3 | 9.5 |
| MLG910-Δ*rpoS* | Stationary | M9+glucose | 1.3 | 13.0 |
| MLG910-Δ*rpoS* | Stationary | M9+glycerol | 1.3 | 13.0 |

For a given set of conditions (strain, growth phase, media, OD_600_ value), the corresponding cell density is listed, as determined by a direct hemocytometer count.
